# Solvent-free copper-catalyzed click chemistry for the synthesis of *N*-heterocyclic hybrids based on quinoline and 1,2,3-triazole

**DOI:** 10.3762/bjoc.13.232

**Published:** 2017-11-06

**Authors:** Martina Tireli, Silvija Maračić, Stipe Lukin, Marina Juribašić Kulcsár, Dijana Žilić, Mario Cetina, Ivan Halasz, Silvana Raić-Malić, Krunoslav Užarević

**Affiliations:** 1 Laboratory for Green Synthesis, Ruđer Bošković Institute, Bijenička 54, HR-10000 Zagreb, Croatia; 2 Department of Organic Chemistry, Faculty of Chemical Engineering and Technology, University of Zagreb, Marulićev trg 20, HR-10000 Zagreb, Croatia; 3 University of Zagreb, Faculty of Textile Technology, Department of Applied Chemistry, Prilaz baruna Filipovića 28a, HR-10000 Zagreb, Croatia

**Keywords:** electron spin resonance (ESR) spectroscopy, in situ Raman monitoring, mechanochemistry, quinoline, solid-state click chemistry

## Abstract

Copper-catalyzed mechanochemical click reactions using Cu(II), Cu(I) and Cu(0) catalysts have been successfully implemented to provide novel 6-phenyl-2-(trifluoromethyl)quinolines with a phenyl-1,2,3-triazole moiety at O-4 of the quinoline core. Milling procedures proved to be significantly more efficient than the corresponding solution reactions, with up to a 15-fold gain in yield. Efficiency of both solution and milling procedures depended on the *p*-substituent in the azide reactant, resulting in H < Cl < Br < I reactivity bias. Solid-state catalysis using Cu(II) and Cu(I) catalysts entailed the direct involvement of the copper species in the reaction and generation of highly luminescent compounds which hindered in situ monitoring by Raman spectroscopy. However, in situ monitoring of the milling processes was enabled by using Cu(0) catalysts in the form of brass milling media which offered a direct insight into the reaction pathway of mechanochemical CuAAC reactions, indicating that the catalysis is most likely conducted on the surface of milling balls. Electron spin resonance spectroscopy was used to determine the oxidation and spin states of the respective copper catalysts in bulk products obtained by milling procedures.

## Introduction

The copper-catalyzed azide–alkyne cycloaddition (CuAAC) represents a prime example of click chemistry. Click chemistry describes “a set of near-perfect” reactions [1] for an efficient regioselective generation of 1,4-disubstituted 1,2,3-triazoles [1–3]. After their discovery [1], click reactions affording 1,2,3-triazoles rapidly became important for simple and robust binding of versatile molecules and for the building of stable polymer structures [4]. At the same time, the 1,2,3-triazoles became the heterocycle of choice in drug discovery, due to their favourable pharmacokinetic and safety profiles, hydrogen-bonding capability, moderate dipole moment, rigidity and stability under in vivo conditions [5–6]. Also, the ability of 1,2,3-triazoles to act as amide bond bioisosteres made the click reaction a valuable synthetic methodology for conjugation of bioactive molecules [7–9] aiming to improve their biological activities [4,10–11]. Discovery of copper(I) ion catalysis in azide–alkyne cycloadditions was decisive for applications of this reaction, as it increases reaction rates and yields and directs the azide–alkyne cycloaddition exclusively towards 1,4-substituted regioisomers, whereas the non-catalyzed process results in a non-stoichiometric mixture of 1,4- and 1,5-regioisomers. Even though CuAAC reactions are efficiently performed in solution, there is a persistent incentive to find greener alternatives, which would reduce time and energy requirements as well as waste generated by these reactions. Among other non-conventional approaches such as microwave and ultrasound irradiation [7,12–13], mechanochemistry has emerged as a viable approach for CuAAC. In a broader sense, mechanochemistry, i.e., chemical transformations induced by mechanical force [14], has been rapidly advancing in various fields of synthesis and materials sciences, including inorganic [15], organic [16–17] and supramolecular materials [18–19], intermetallic compounds [20], nanoparticles [15,21], and with a wide application in the synthesis of pharmaceutical solids [22]. Furthermore, medicinal mechanochemistry, a new research discipline that provides an access to the active pharmaceutical ingredients, is anticipated to have a strong impact on the future development of medicinal chemistry and demands of the pharmaceutical industry for greener and more efficient approaches to chemical synthesis [23–25]. In accordance with the progress of mechanochemistry in organic syntheses [26], ball milling has been successfully implemented for solvent-free CuAAC reactions [27–30]. Significantly shortened reaction time and reduced energy requirements, along with clear benefits in yields revealed a wide potential of the mechanochemical approach for CuAAC. The initial report showed applications of standard catalyst systems, copper(II) salts and ascorbic acid [27], but it was soon demonstrated that the application of mechanochemistry allowed for the use of heterogeneous copper(0) catalysts, either as copper milling vessels [28] or copper powder [30] for performing CuAAC rapidly and efficiently. The use of a copper(0) catalyst for CuAAC is also known in solution, but these reactions are usually much slower [31]. Also, click polymerization was applied using a ball-milling process with no significant influence on the integrity of the polymer chain [27,32].

Herein we have studied the efficiency of copper catalysts with Cu(0), Cu(I) and Cu(II) oxidation states for the mechanochemical CuAAC reaction of target quinoline derivatives and *p*-substituted phenyl azides. We have also investigated the effect of the *p*-substituent in the azide on the reaction progress and yields. Direct monitoring by in situ Raman spectroscopy was used to gain an insight into the milling CuAAC reaction pathway when using different catalysts. The electronic structure of Cu catalysts after the reaction completion was assayed by electron spin resonance (ESR) spectroscopy. All milling reactions, except the one using copper(0) as catalyst, were compared to solution procedures to establish the benefits of each synthetic method. The structures of all products were determined by single-crystal X-ray diffraction, and the products were additionally characterized by NMR, Raman and FTIR–ATR spectroscopic methods.

## Results and Discussion

### Conventional solution-based click reactions for the synthesis of **5–8**

**Scheme 1:**
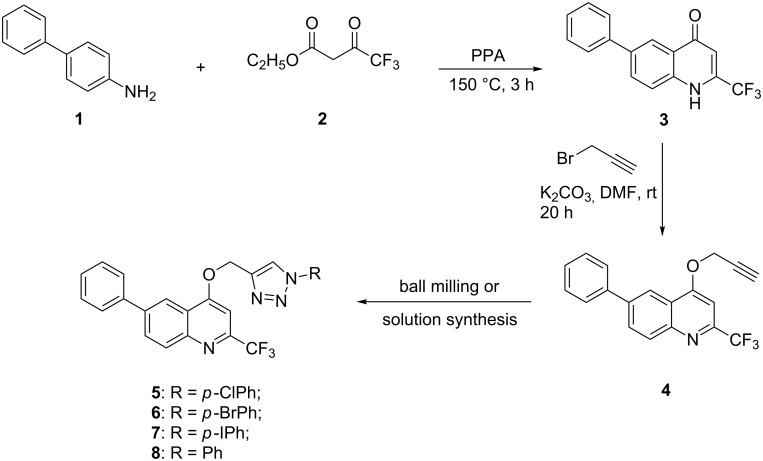
Synthetic procedures for preparation of *p*-halogen-substituted and non-substituted phenyl-1,2,3-triazole 6-phenyl-2-(trifluoromethyl)quinolines.

Based on the recently obtained 1,2,3-triazole-appended *N*-heterocycles, as promising lead compounds with efficient and selective cytostatic activities [8–9], our research groups share an interest in derivatization of target compounds by a triazole bridge [33]. Quinoline is an important constituent of compounds with diverse applications, some of which display potent cytostatic activity through different mechanisms of action such as DNA intercalation, apoptosis, abrogation of cell migration, inhibition of angiogenesis and disregulation of nuclear receptor signaling [34–35]. Moreover, it was found that halogenated compounds have an important role in therapeutic application increasing their lipophilicity, metabolic stability and improving interactions of protein–ligand complexes [36]. Taking into consideration the aforementioned, we have designed and synthesized 6-phenylquinoline derivatives containing a trifluoromethyl group at C-2 and a *p*-halogen-substituted and non-substituted phenyl-1,2,3-triazole moieties. The synthesis of 2-(trifluoromethyl)-6-phenylquinolone was achieved by Conrad–Limpach reaction of a primary aromatic amine with a β-ketoester [37–38]. Namely, thermal condensation of 4-aminobiphenyl (**1**) with ethyl 4,4,4-trifluoro-3-oxobutanoate in polyphosphoric acid (PPA) followed by the cyclization of the Schiff base intermediate afforded the 2-(trifluoromethyl)-6-phenylquinolone **3** (Scheme 1).

*O*-Alkynylquinoline derivative **4** required for the click synthesis of target triazoles was obtained in the second step using propargyl bromide in the presence of K_2_CO_3_, as a base, to afford exclusively the *O*-substituted quinoline, with no traces of the *N*-substituted analog. The formation of the *O*-propargyl regioisomer was confirmed by NMR spectroscopy using the connectivity between *O*-methylene and methine C-3 protons displayed in a ^1^H,^1^H-NOESY spectrum of **4** (Figure S10 in Supporting Information File 1). Compound **4** was then submitted to Cu(I)-catalyzed 1,3-dipolar cycloaddition with selected halogen-substituted and non-substituted aromatic azides to yield target *N*-heterocyclic hybrids **5**–**8** containing quinoline and 1,2,3-triazole scaffolds. Based on the known protocols for click conjugation [39] that include direct utilization of a Cu(I) source as well as alternative creation of Cu(I) from a Cu(II) source or elemental copper, initially we have examined the most common CuAAC reaction procedure using in situ generated Cu(I) through the reduction of Cu(II).

Conventional solution-based CuAAC reaction using copper(II) acetate monohydrate was applied to provide triazoles **5**–**8**. Two modes of heating the reaction mixture were used in order to test the reactivity of the azide reactants: heating at 60 °C for 3.5 h (method 1a) and heating at 60 °C overnight (method 1a^*^). Reaction with *p*-iodophenyl azide, which furnished the target compound **7**, was the most efficient giving the same high yield (89%) performed either by method 1a or method 1a*, Table 1, entry 3.

**Table 1:** Reaction conditions and yields for the solvent-free mechanochemical and solvent-based conventional click reactions to afford 1,4-disubstituted 1,2,3-triazole **5**–**8**.

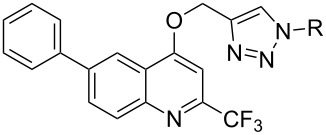 **5–8**						

Entry	Compound	R	Conventional click reaction	Yield [%]^a^	Mechanochemical click reaction	Yield [%]^a^

1	**5**	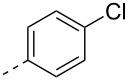	method 1a method 1b method 1a^*^	21 5 77	method 2a method 2b method 2c	57 85 77
2	**6**	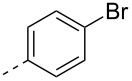	method 1a method 1b method 1a^*^	45 40 76	method 2a method 2b method 2c	60 87 80
3	**7**	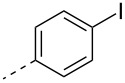	method 1a method 1b method 1a^*^	89 52 89	method 2a method 2b method 2c	77 92 87
4	**8**	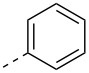	method 1a method 1b method 1a^*^	10 5 21	method 2a method 2b method 2c	72 79 76

^a^Yields were determined after isolation of product using column chromatography. Conventional click reaction. Method 1a: Cu(OAc)_2_·H_2_O, CH_3_OH, 60 °C, stirring for 3.5 h; method 1a^*^: Cu(OAc)_2_·H_2_O, CH_3_OH, 60 °C, stirring overnight; method 1b: CuI, DIPEA, acetic acid, CH_2_Cl_2_, rt, 3.5 h stirring. Mechanochemical click reaction. Method 2a: Cu(OAc)_2_·H_2_O, two stainless-steel milling balls (7 mm), PTFE vessel, 3.5 h, rt, 30 Hz; method 2b: CuI, DIPEA, acetic acid, two stainless-steel milling balls (7 mm), PTFE vessel, 3.5 h, rt, 30 Hz; method 2c: DIPEA, acetic acid, PTFE vessel, two brass balls (7 mm), rt, 3.5 h.

However, the isolated yields were significantly raised by application of method 1a* for the *p*-chloro- (from 21 to 77%, Table 1, entry 1) and *p*-bromophenyl azides (from 45 to 76%, Table 1, entry 2). On the other hand, the reaction with the non-substituted azide in all solution procedures, even by method 1a*, gave compound **8** in low yield (5–21%, Table 1, entry 4). Solution-based method 1b using CuI, *N*,*N’*-diisopropylethylamine (DIPEA) and acetic acid afforded compounds **5**–**7** in 5–52% isolated yield and was thus less successful for the synthesis of **5**–**8** derivatives than methods 1a and 1a*, which include copper(II) acetate monohydrate as catalyst. Methods 1a and 1a*, however, include heating of reaction mixture to 60 °C, so the methods 1a and 1b are not readily comparable.

The efficiency of triazole formation using the method 1b steadily grows from a yield of 5% for the non-substituted azide (entry 4, Table 1) to ca. 50% for the *p*-iodo-substituted azide (entry 3, Table 1), resulting in the following order of reactivity: H < Cl < Br < I. These results are somewhat contrary to common CuAAC which are considered to be insensitive to electronic properties of both the alkyne and the azide [40]. It is evident here that the solution reaction with the azide bearing the iodo substituent resulted in almost 10-fold better yield in comparison to that of the unsubstituted azide (Table 1). When considering the proposed mechanism for CuAAC [3,41], such an influence of the electronic structure of the azide reactant could be tentatively ascribed to a reaction step where the azide is coordinated to the copper–alkyne complex via the most negative nitrogen (the one closest to the phenyl ring), before proceeding to the cyclization step with the coordinated alkyne.

### Mechanochemical click reactions for the synthesis of **5–8**

In order to investigate the eficiency of different copper species for the solvent-free mechanochemical CuAAC in a ball mill, we conducted a number of milling experiments where we assayed catalytic action of most commonly used copper(0), copper(I) and copper(II) catalysts. Mechanochemical reactions were compared to traditional solvent-based procedures, except for CuAAC with the Cu(0) catalyst, which was reported to be very slow in solution [31]. Various synthetic approaches used here are described in detail in the Experimental section and briefly in Table 1, where a comparison between solution-based and milling syntheses using different copper catalysts is given.

Milling using copper(II) acetate monohydrate (method 2a) was performed without a reducing agent. The Cu(II) catalyst proved effective for mechanochemical CuAAC, affording pure **5–8** in 60–80% isolated yield. Using copper(I) iodide as the catalyst in the presence of *N,N*-diisopropylethylamine (DIPEA) (method 2b) significantly increased yields for each respective CuAAC process, yielding up to 92% of the isolated triazole product (entry 3, Table 1), with the ^1^H NMR spectra of the reaction mixture showing complete conversion of the reactants. Method 2b was additionally tested in the absence of DIPEA, which lowered the yield of the reactions by 10–20% points. It is well documented that the presence of DIPEA increases the yield of CuI-catalyzed CuAAC in solution [42], due to its role in the deprotonation of the alkyne substrate and easier formation of the reactive Cu(I) acetylide intermediate [3,42]. We continued to study mechanochemical CuAAC reactions by introducing copper(0) to the reaction mixture using copper milling vessels. Leaching and wearing of milling vessels or balls during the milling process was an object of several studies [43–44], and Mack and co-workers found how to exploit it for catalytic purposes. They manufactured copper milling equipment as catalysts for mechanochemical CuAAC [28], resulting in good to excelent yields of the studied CuAAC reactions. It was recently shown how even the addition of simple copper powder to the reaction mixture can be successfully used for the mechanochemical CuAAC process [30]. In our case, however, using copper milling vessels did not result in good reaction yields (less than 20%), and the product was littered with copper microparticles. As an alternative to copper vessels, we have tested vessels made from brass, an alloy of copper and zinc, which is much harder and mechanically more resistant than pure copper. We tested two approaches, one using a completely brass milling assembly (brass milling vessels and balls), while the other combined brass milling balls with polytetrafluoroethylene (PTFE, Teflon) vessels. Surprisingly, using brass milling equipment did not increase the yields of the studied click rections, which still remained bellow 25%. In an attempt to activate the brass, as a catalyst, we added DIPEA and a small amount of acetic acid to the reaction mixture. Such an improvement of the synthetic procedure resulted in complete conversions of reactants to the triazole products with the isolated yields ranging from 80–90%. After the isolation and purification, copper-sensitive ESR spectroscopy showed no traces of copper in the products (Materials and methods within the Experimental section).

Compared to solution procedures, CuAAC reactions proved to be more efficient under solvent-free ball-milling conditions, with ca. 15-fold increase in yields of products **5** and **8**. Tested mechanochemical methods showed the same dependence of reactivity to the *p*-substituent as reactions in solution, H < Cl < Br < I, but the difference in yields was significantly less pronounced.

### In situ Raman monitoring of mechanochemical click reactions

In an attempt to gain a direct insight into reaction pathways of mechanochemical CuAAC reactions we repeated milling experiments 2a–2c in the preparation of the chloro-substituted product **5** while monitoring the reaction course by in situ Raman spectroscopy [45]. While this methodology was already successfully applied for establishing mechanistic and kinetic details in the formation of cocrystals [46], coordination and organometallic compounds [47], it proved to be especially valuable for the organic solid-state synthesis, revealing the base-catalysis in an amide formation reaction [48], and detecting intermediate phases not available from solution [49].

**Figure 1:**
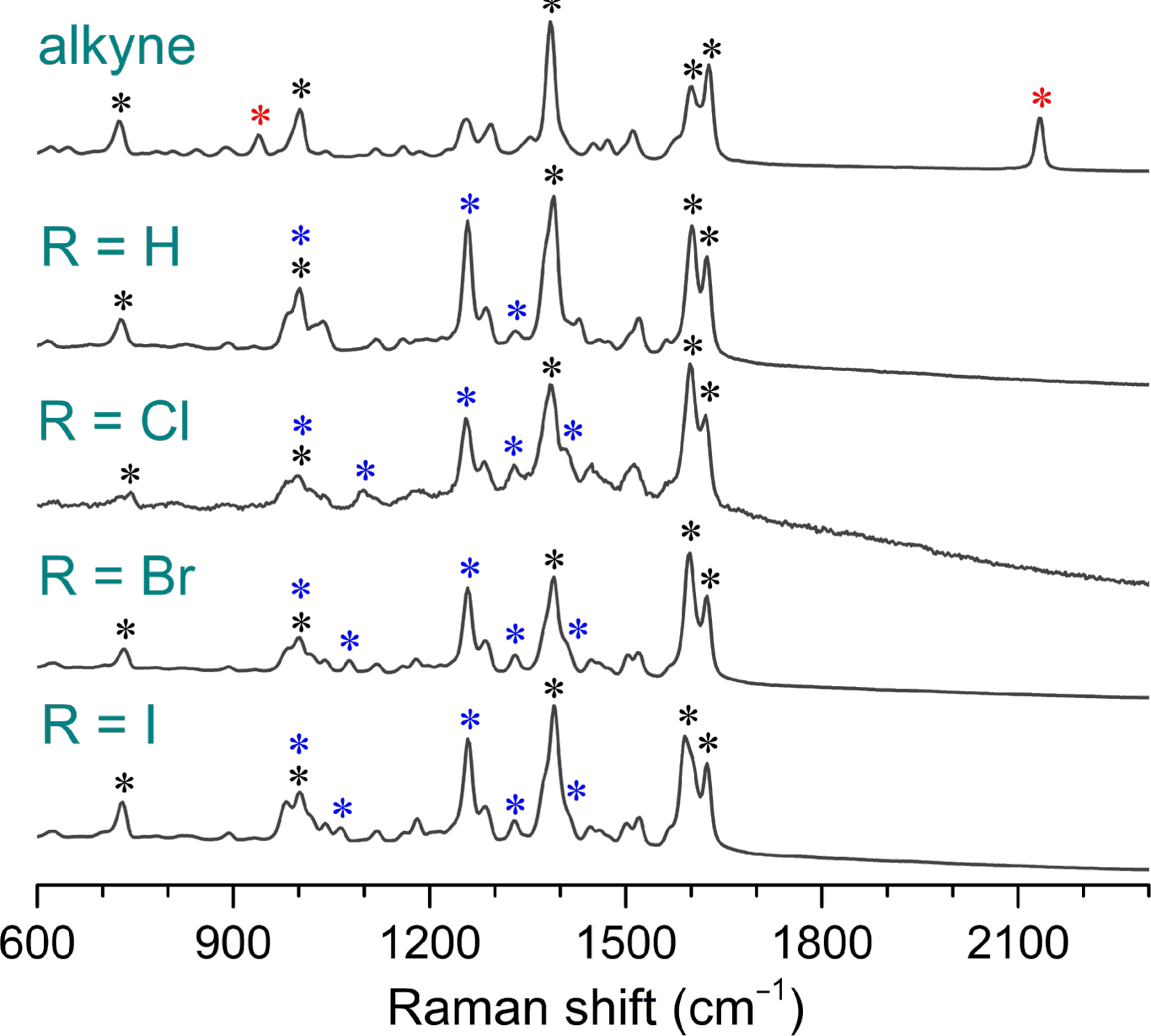
Experimental Raman spectra of the alkyne **4** and triazole products **5**–**8**. Bands attributed to the vibrational modes common to all compounds are marked with a black asterisk (*). Bands assigned to the alkyne and triazole products are marked with red and blue asterisks, respectively. For detailed vibrational analysis of these compounds please refer to Table S1, Supporting Information File 1.

Raman spectra (Figure 1) were assigned combining literature data [50] and DFT calculations.

Calculated spectra are shown in Figures S15–S19 in Supporting Information File 1. Raman spectra of all studied compounds, the alkyne **4** and the isolated products **5**–**8**, are characterized by strong bands assigned to various vibrations of aromatic rings (Figure 1 and Supporting Information File 1, Table S1). Dried aryl azides were excluded from measuring due to their explosive nature (Materials and methods within the Experimental section). According to calculations, vibrations of all rings contribute to two bands at about 1600 cm^−1^ as well as bands at 1000 and 730 cm^−1^, whereas stretching vibrations including the quinoline C(9)–C(10) bond dominantly contributes to a strong band about 1360 cm^−1^. Raman spectrum of the alkyne reactant contains a fingerprint medium intensity band at 2133 cm^−1^ assigned to stretching of the triple C≡C bond.

Solid triazole products have mutually similar Raman spectra as the only significant structural difference is a *p*-substituent on the phenyl ring originating from the azide reactant. Apart from the phenyl and quinolinyl vibrations, a strong band observed at 1258 cm^−1^ is attributed mostly to stretching of the N_3_ group in the triazole ring. Structural diversity in products is supported by observations of weak bands at 1099 (Cl), 1077 (Br) and 1064 (I) cm^−1^ which are assigned to vibration of the phenyl ring that contains the carbon–halogen bond. Characteristic C≡C alkyne band at 2133 cm^−1^ along with the band at 1258 cm^−1^ of the triazole products are appropriate for monitoring of the reaction progress.

**Figure 2:**
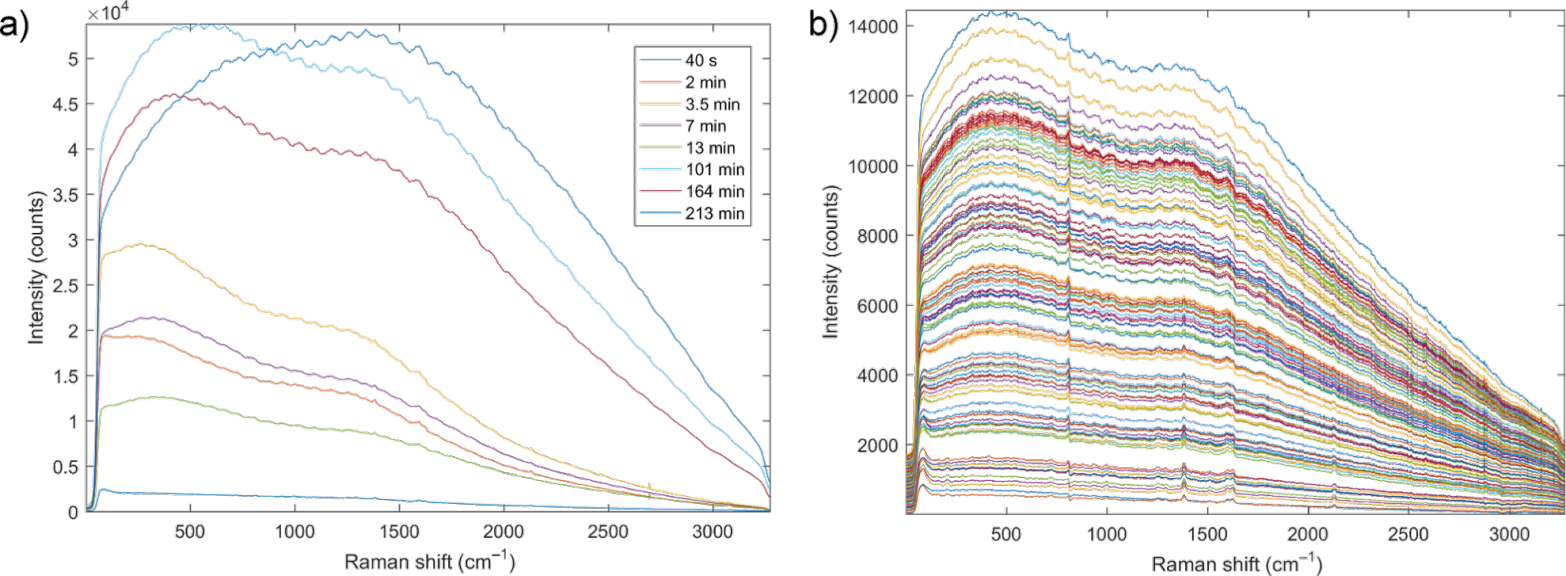
In situ Raman monitoring of a) mechanochemical formation of triazole **5** using copper(II) acetate monohydrate as catalyst (method 2a); and b) mechanochemical formation of triazole **5** by method 2b using CuI/DIPEA catalyst.

In situ Raman monitoring of formation of the triazole **5** using copper(II) acetate monohydrate (5 mol %, method 2a) revealed strong luminescence of the reaction mixture indicating the direct involvement of the catalyst in the milling process and the formation of luminescent copper species, which hindered a detailed insight into the reaction pathway. Nevertheless, the starting Raman spectrum had a clearly visible alkyne signal, which was, however, after a couple of minutes milling, covered by two broad luminescent “humps”, Figure 2a.

After 13 minutes milling no pronounced Raman bands could be unambiguously detected. The luminescence of the reaction mixture gradually changed during milling and the final spectrum after 213 minutes milling exhibited a single luminescent maximum centered at around 1500 cm^−1^ (Figure 2a) possibly due the formation of different copper complexes as milling progressed. Milling by method 2b, where the catalyst CuI was added in concentrations of 2 mol %, showed strong luminescence similar to the one observed in milling by method 2a, starting after ca. 3 minutes milling and covering most of Raman signals already after 10 minutes milling. In this case, however, luminescence grew steadily but the positions of the two luminescent peaks did not change until the end of milling (Figure 2b). While milling using CuI alone did not result in raise of luminescence (Figure S20a in Supporting Information File 1), growth of the luminescent peak was observed when the CuI was milled with the purified triazole product **5**, indicating the interaction between CuI and **5** that occurred during the milling process (Figure S20b in Supporting Information File 1). Here, the two broad luminescent “humps” with position similar to those observed with method 2b prevented clear detection of Raman vibrations even after 15 minutes milling.

**Figure 3:**
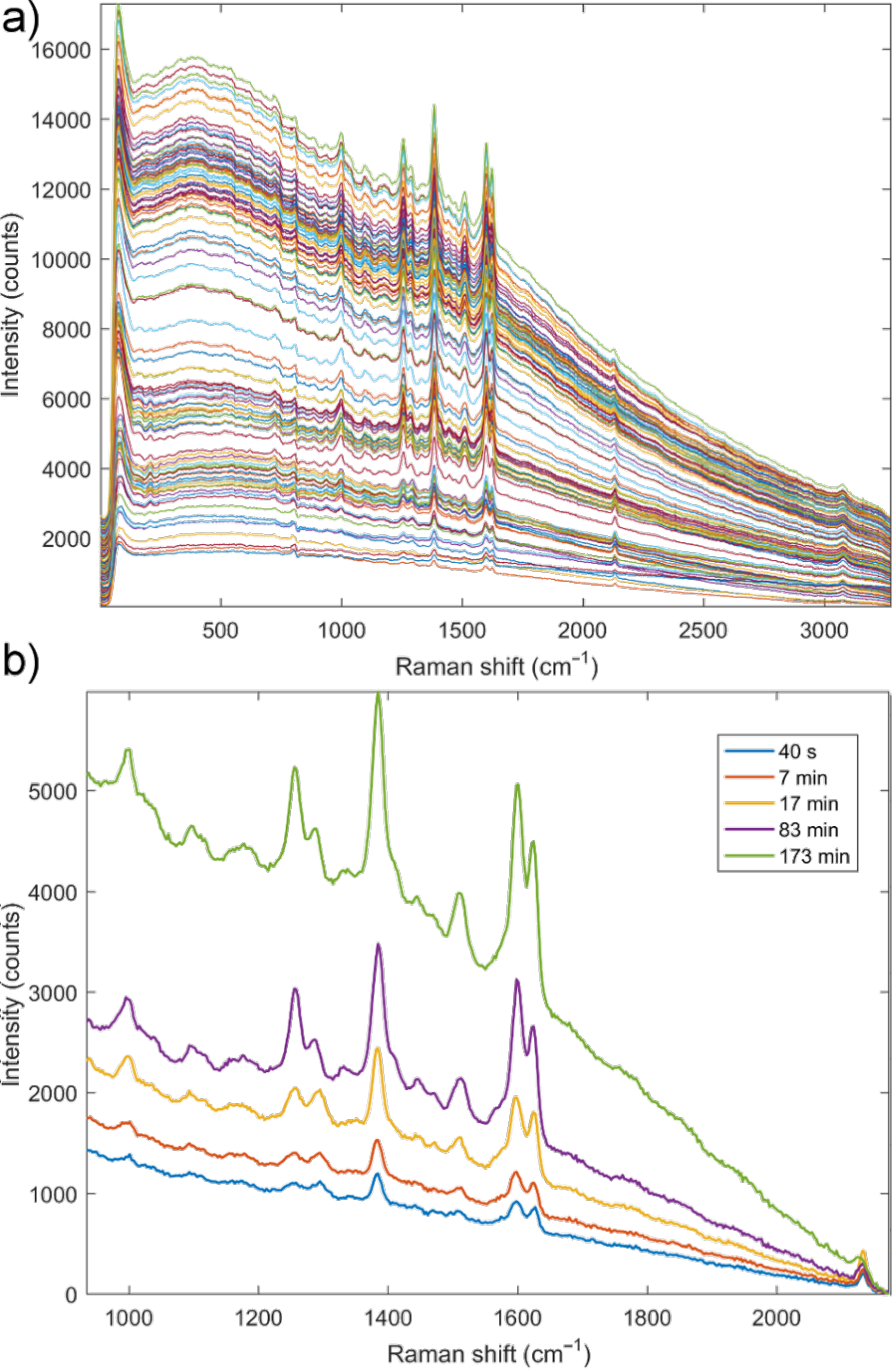
a) In situ Raman monitoring for mechanochemical synthesis of **5** using brass balls and PMMA reaction vessel. b) Selected Raman spectra from panel a) highlighting the slow transformation of the alkyne to the triazole product. The characteristic C≡C alkyne band at 2133 cm^−1^ along with the triazole band at 1258 cm^−1^ of the triazole product (Supporting Information File 1, Table S1) are suitable to evaluate the reaction progress. The C≡C band is still visible after 210 minutes milling, indicating that the reaction was not complete.

Surprisingly, monitoring the mechanochemical formation of **5** by milling with brass balls (method 2c) enabled a clear insight into the evolution of the reaction mixture (Figure 3a). The luminescent peak remained weak throughout the experiment, leaving the Raman signals of the reaction participants clearly visible.

Analysis of time-resolved Raman monitoring data showed a direct formation of the product **5**, without any detectable intermediates. The C≡C band was very weak but still visible at the end of the milling, indicating that 210 minutes milling was not enough to complete this reaction, which was further corroborated by ex situ analyses. The fact that we were able to monitor milling by method 2c, as opposed to methods 2a and 2b where copper catalyst was directly added to reaction mixture in catalytic quantity of 2–5 mol %, could tentatively be explained by even a lower content of copper compounds in the reaction mixture. This strongly indicates that during mechanochemical reactions with milling balls containing copper(0), the catalytic process is mostly happening on the surface of milling balls, and diffusion of copper ions to reaction mixture is minute. This could further explain the absence of other intermediate species in the spectra of solid reaction mixture, such as copper–alkyne complexes, which are commonly considered as a part of the solution catalytic cycle [51]. We anticipate that monitoring these highly luminescent CuAAC reactions by using advanced Raman techniques such as shifted-excitation Raman difference spectroscopy (SERDS) could be possible [52]. In this way, mechanistic details of these reactions and the behavior of all studied copper catalysts may be more visible, opening the path towards elucidation of mechanism(s) for the solvent-free click reactions.

### Electron spin resonance (ESR) spectroscopy

**Figure 4:**
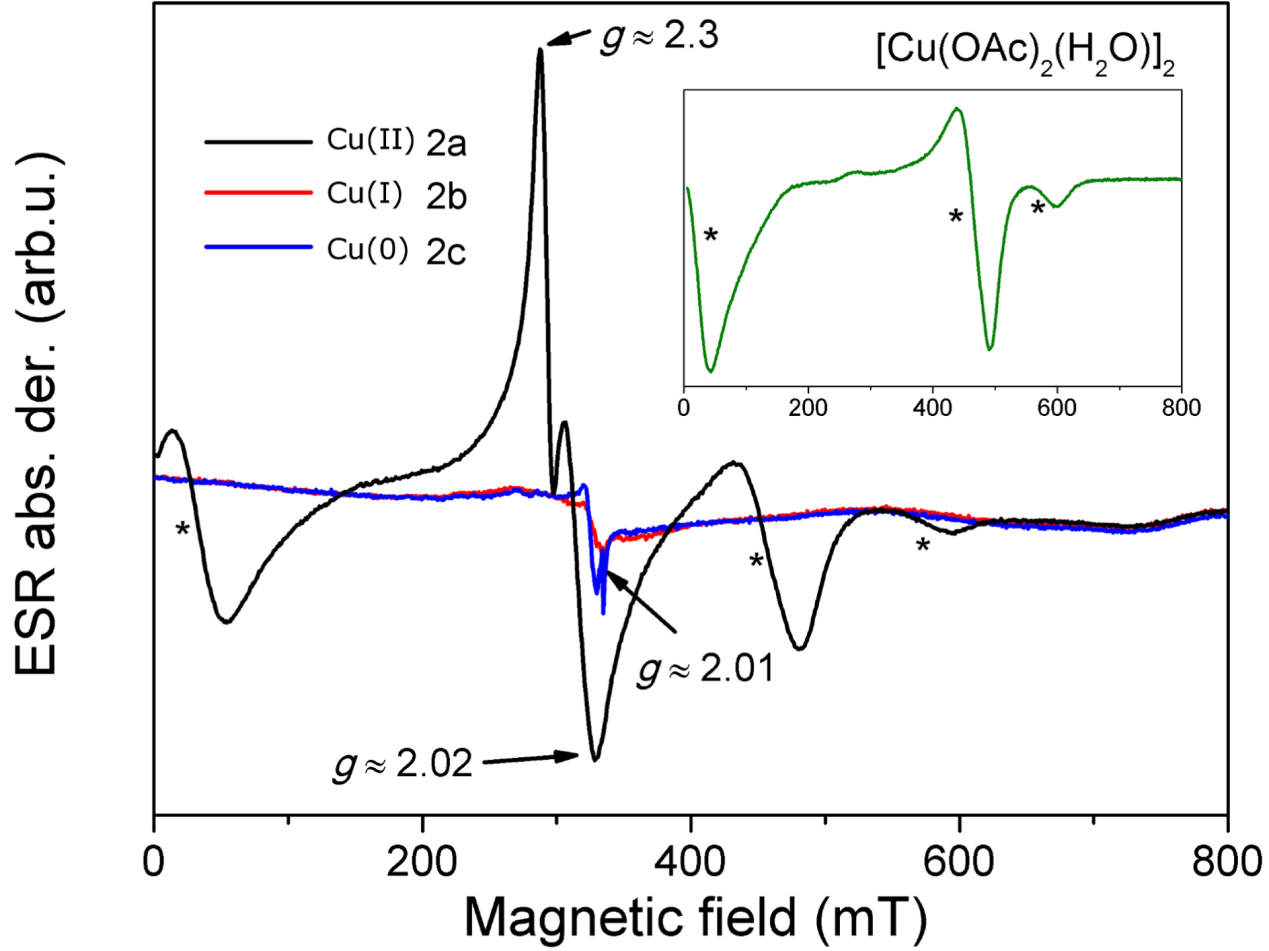
ESR spectra of samples obtained after milling by methods 2a (black), 2b (red) and 2c (blue). The inset shows the spectrum of [Cu(OAc)_2_(H_2_O)]_2_ [53]. All spectra are recorded at room temperature.

ESR is an ideal technique for validating the oxidation and spin state of copper cations. Elemental copper and copper(I) are ESR silent, whereas the copper(II) shows strong and characteristic lines revealing local properties of this ion. Here we were interested to establish how the milling procedures 2a–2c for the synthesis of **5** would affect the oxidation state and coordination modes of all three evaluated catalysts when the milling was performed in air. Analyzing the reaction mixture after milling with brass balls (method 2c, DIPEA and acetic acid added) showed that there are no copper(II) cations present in the final mixture (Figure 4). The ESR spectrum reveals only the presence of free radicals, characterized by sharp signal with *g*-value *g* ≈ 2.01.

Milling the azide and alkyne with copper(I) catalytic system (CuI/DIPEA/acetic acid, method 2b) resulted in an ESR silent yellow product, revealing that the oxidation did not occur and no copper(II) was present in the reaction mixture. To test the sensitivity of CuI to milling in air, we conducted two additional experiments. When the sole CuI was milled for 30 minutes in air, no Cu(II) was detected in the mixture. However, milling the CuI/DIPEA/acetic acid catalytic system as used in method 2b, only without the azide and alkyne reactants, results in oxidation of Cu(I) to Cu(II), with the final product showing ESR lines characteristic for copper(II) acetate. Thus, it seems that the presence of alkyne and azide in the reaction mixture stabilizes the copper(I) ion in its catalytically active state.

The product yielded by method 2a, where copper(II) acetate monohydrate was added as catalyst in 5 mol % quantity, shows a complex ESR spectrum (Figure 4). Three lines marked by asterisks are characteristic for copper(II) acetate monohydrate [53]. These lines reveal the presence of two strongly antiferromagnetically coupled copper ions with spin *S* = 1/2. In the spectrum of the product obtained by method 2a, an additional strong signal is detected (peaks at *g* = 2.02 and *g* = 2.3) that could be assigned to the presence of non-coupled paramagnetic Cu(II) ions in the sample, suggesting that beside the copper(II) acetate paddlewheel complex at least one other copper(II) coordination complex with monomeric core is present in the reaction mixture. Thus, it seems that reacting copper(II) with vast excess of alkyne and azide reactants does not result in the total reduction of copper(II) to the catalytically active form, which can possibly explain the lower efficiency of method 2a in comparison to the other used mechanochemical methods. It should be noted here that the same product after purification by column chromatography shows no traces of copper in the ESR spectrum (Supporting Information File 1, Figure S21).

### X-ray crystal structure analysis

**Figure 5:**
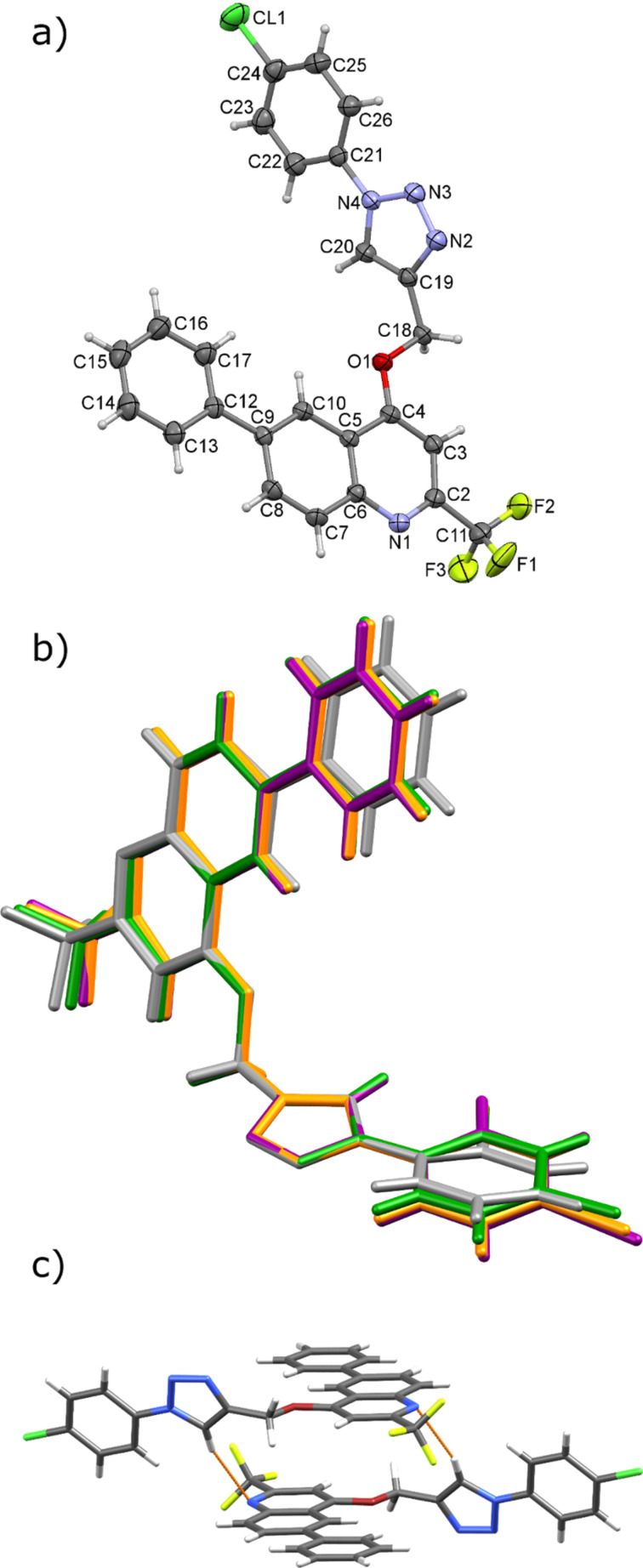
X-ray structure of the triazole compounds. (a) Molecular structure of **5**, with the atom-numbering scheme. Displacement ellipsoids for non-hydrogen atoms are drawn at the 30% probability level. Only the major component of disordered fluorine atoms is presented. (b) Overlap of molecules **5**–**8** showing almost identical molecular conformation. Color code: **5** green, **6** orange, **7** purple, **8** gray. c) Capped-stick representation of **5**, showing the dimer formed by C–H∙∙∙N hydrogen bond (orange stippled lines).

Single-crystal X-ray structure analysis was performed for all products. It provided clear identification of the novel triazole derivatives and it was largely helpful for calculating the Raman spectra for monitoring purposes. It corroborated the substitution of the phenyl-1-(1,2,3-triazolyl)methyl unit at O-4 position of the quinolone heterocycle and formation of the 1,2,3-triazole ring in compounds **5–8** (Figure 5 and Supporting Information File 1, Figure S22). Thus, the molecular structures differ in the substituent bonded to the C24 atom of the C21–C26 phenyl ring, which is chlorine in **5**, bromine in **6**, iodine in **7**, and hydrogen in **8**. The corresponding bond lengths in these structures are similar, as well as the conformations of the molecules (Figure 5b and Supporting Information File 1, section 7).

Compound **5** may serve as a model for the crystal structure description. The molecules of **5** are linked by one C–H∙∙∙N hydrogen bond, so forming a dimer via eighteen-membered ring (e.g., see Figure 5c for **5**) which can be described by graph-set notation as R_2_^2^(18) [54]. Although the same motif formed by the analogous hydrogen bond is observed in other three structures (Table S3, Supporting Information File 1), the final supramolecular structures of **5**–**8** differ, from one-dimensional chains to three-dimensional network. It should be mentioned that the interactions between the present halogen atoms were not observed. For more detailed description of crystal structures of **5**–**8** please refer to the section 7 of Supporting Information File 1 and Figures S23–S28 therein.

## Conclusion

In conclusion, mechanochemistry was successfully applied in CuAAC click reaction to provide the target 6-phenyl-2-(trifluoromethyl)quinolines containing *p*-halogen-substituted and non-substituted phenyl-1,2,3-triazole unit attached at the O-4 position of the quinoline fragment. All triazole products have almost identical conformations in the solid state, with no halogen bonding observed in their crystal structures. Milling procedures using Cu(II), Cu(I) and Cu(0) catalysts proved to be significantly more efficient than the corresponding solution reactions, with up to 15-fold gain in yield. Both procedures showed the same reactivity trend, resulting in the H < Cl < Br < I bias, but the differences in yields for solution procedures were much more pronounced. In situ Raman monitoring of the milling processes using Cu(I) and Cu(II) catalysts revealed active involvement of copper catalysts through coordination and occurrence of strongly luminescent copper compounds which, despite the fact they were present in mere 2–5 mol %, completely covered vibrational Raman bands. On the contrary, using copper(0) in the form of brass milling balls resulted in a mild luminescence of the reaction mixture and enabled a direct insight into the reaction pathway, which showed direct transformation of reactants to products. Thus, we propose that the catalytic reaction for the method 2c is most likely occurring on the surface of brass milling balls, with minute diffusion of the copper ions to the reaction mixture. During the milling reactions, copper(0) and copper(I) catalysts do not oxidize to Cu(II) when the alkyne and azide are present in the reaction mixture, while in the product obtained after the milling with copper(II) catalyst (5 mol %) a significant amount of copper(II) ions are still present. In future, we will be focused on elucidating the solid-state mechanisms for this important class of organic reactions by applying advanced in situ Raman monitoring techniques. Screening of cytostatic and antibacterial activities of novel compounds **5**–**8** and their structural analogs will be reported in due course.

## Experimental

**Materials and methods*.*** Compounds **5**–**8** were synthesized from corresponding aryl azides (0.5 M in *tert*-butyl methyl ether, ≥95.0%) that were obtained commercially from Sigma-Aldrich. To ensure solvent-free milling conditions, *tert*-butyl methyl ether was evaporated under vacuo immediatelly before the milling was commenced. The progress of reactions was monitored using thin-layer chromatography (TLC) on pre-coated Merck silica gel 60F-254 plates with an appropriate solvent system and the spots were detected under UV light (254 nm). Column chromatography was performed using silica gel (Fluka, 0.063–0.2 mm). In order to scavenge the copper residues from the click reactions, one additional column chromatography using aluminium oxide (Fluka, 0.063–0.2 mm) was performed. Melting points (uncorrected) were determined with a Kofler micro hot-stage (Reichert, Wien) apparatus.

NMR spectra were acquired on a Bruker 300 and 600 MHz NMR spectrometer. Spectra were recorded in DMSO-*d*_6_ at 298 K. Chemical shifts were referenced to the residual solvent signal of DMSO at δ 2.50 ppm for ^1^H and δ 39.50 ppm for ^13^C. Individual resonances were assigned on the basis of their chemical shifts, signal intensities, multiplicity of resonances and H–H coupling constants (Supporting Information File 1, Figures S1–S5, S10).

High-resolution mass spectra of the final compounds were recorded on Applied Biosystems 4800 Maldi TOF/TOF Analyzer (Supporting Information File 1, Figures S6–S9).

Mechanochemical reactions were carried out using an IST500 (InSolido Tehnologies, Croatia) mixer mill operating at 30 Hz in PTFE reaction vessels using stainless steel or brass balls.

Fourier-transform infrared attenuated total reflectance spectroscopy (FTIR–ATR) was performed using a Perkin-Elmer SpectrumTwo spectrometer, from 4400 cm^−1^ to 500 cm^−1^, with resolution 4 cm^−1^ (Supporting Information File 1, Figures S11–S14).

Computational details. Calculations were carried out using the B3LYP hybrid functional combined with an empirical Grimme’s D3 dispersion correction [55] (B3LYP-D3) implemented in Gaussian 09 [56]. The standard 6-311+G(2d,p) basis set with the ultrafine method was used for C, H, N, F, Cl and Br atoms. Iodine atoms were modeled by the Stuttgart−Dresden (SDD) pseudopotential and the accompanying SDD basis set [57]. Full geometry optimization in the gas phase was followed by vibrational frequency calculations that identified calculated stationary points as minima. Calculated Raman spectra were scaled by 0.98 (Supporting Information File 1, Figures S15–S19, Table S1).

In situ Raman monitoring of mechanochemical reactions was performed in translucent and amorphous reaction vessels made from poly(methyl metacrylate) (PMMA) using a portable Raman system with a PD-LD (now Necsel) BlueBox laser source (excitation wavelength 785 nm) equipped with B&W-Tek fiber optic Raman BAC102 probe, and coupled with Maya2000Pro (OceanOptics) spectrometer. The probe was positioned under the milling vessel using a movable stand, so to place a focus of the laser ≈1 mm inside of the vessel.

ESR spectroscopy was performed on a Varian E-9 spectrometer, at room temperature. The measurements were obtained at the microwave frequency around 9.3 GHz with the magnetic field modulation amplitude of 0.5 mT. For detecting copper in the final products, ESR spectra were recorded by an X-band Bruker Elexsys 580 FT/CW spectrometer with a microwave frequency around 9.7 GHz. The measurements were performed at a modulation frequency of 100 kHz and a magnetic field modulation amplitude of 0.5 mT. The results are shown in Supporting Information File 1, Figure S21.

X-ray crystal structure analysis. Single crystals of **5**–**8** suitable for single crystal X-ray structure analysis were obtained at room temperature by partial evaporation of the solvent from the mixture of dichloromethane and methanol. Data for **5**–**7** were collected at 295 K on a Oxford Diffraction Xcalibur2 diffractometer with a Sapphire 3 CCD detector using graphite-monochromatized Mo K*_α_* radiation (λ = 0.71073 Å). Data for **8** were collected at the same temperature on Oxford Diffraction Xcalibur Nova R diffractometer with Ruby detector using mirror-monochromatized Cu K*_α_* radiation (λ = 1.54184 Å). The *CrysAlisPro* program [58] was used for the data collection and processing. The intensities were corrected for absorption using the multi-scan absorption correction method (**5**, **7** and **8**) and gaussian absorption correction method (**6**) [58]. All structures were solved using direct methods with SIR–2004 [59] and refined by full-matrix least-squares calculations based on *F*^2^ using SHELXL–2016 [60] integrated in the WinGX program package [61]. All hydrogen atoms were included in calculated positions, with SHELXL–2016 defaults. Fluorine atoms of trifloromethyl groups in **5**–**8** were disordered and have been refined with fixed occupancy ratio of 0.60/0.40 in **5** and **8**, 0.70/0.30 in **6**, and 0.68/0.32 in **7**. Geometric restraint on some of the C–F distances and restraint on anisotropic displacement parameters of some fluorine atoms in **5**–**8** were applied in the refinement. The PLATON [62] and Mercury [63] programs were used for structure analysis and molecular and crystal structure drawings preparation. The CCDC 1549136-1549139 contain the supplementary crystallographic data for this paper. These data can be obtained free of charge from The Cambridge Crystallographic Data Centre via http://www.ccdc.cam.ac.uk/data_request/cif.

Crystal data for **5**: 0.763 × 0.424 × 0.155 mm^3^; C_25_H_16_ClF_3_N_4_O, *M*_r_ = 480.87, triclinic, space group *P*-1 (No. 2); *a* = 8.0775(4) Å, *b* = 10.3530(5) Å, *c* = 13.7751(6) Å, α = 82.383(4)°, β = 74.062(4)°, γ = 84.946(4)°, *V* = 1096.29(9) Å^3^; *Z* = 2; ρ = 1.457 g cm^−3^, μ(Mo K_α_) = 0.226 mm^−1^; θ_max_ = 27.999°, 19408 reflections measured, 5276 unique reflections and 3932 with *I* ≥ 2σ(*I*), *R*_int_ = 0.0337; Final *R* indices [(*I* > 2σ(*I*)]: *R* = 0.0538, *wR* = 0.1453, [all data]: *R* = 0.0729, *wR* = 0.1603, *S* = 1.180 for 334 parameters and 23 restraints, largest diff. peak and hole 0.335/−0.403 *e* Å^−3^.

Crystal data for **6**: 0.774 × 0.563 × 0.335 mm^3^; C_25_H_16_BrF_3_N_4_O, *M*_r_ = 525.33, triclinic, space group *P*-1 (No. 2); *a* = 8.0114(7) Å, *b* = 10.5132(8) Å, *c* = 13.8073(11) Å, α = 93.316(6)°, β = 105.865(7)°, γ = 94.002(6)°, *V* = 1112.31(16) Å^3^; *Z* = 2; ρ = 1.568 g cm^−3^, μ(Mo K_α_) = 1.899 mm^−1^; θ_max_ = 27.999°, 13486 reflections measured, 5351 unique reflections and 2785 with *I* ≥ 2σ(*I*), *R*_int_ = 0.0622; Final *R* indices [(*I* > 2σ(*I*)]: *R* = 0.0614, *wR* = 0.1418, [all data]: *R* = 0.1300, *wR* = 0.1778, *S* = 1.056 for 334 parameters and 35 restraints, largest diff. peak and hole 0.408/−0.733 *e* Å^−3^.

Crystal data for **7**: 0.871 × 0.660 × 0.330 mm^3^; C_25_H_16_F_3_IN_4_O, *M*_r_ = 572.32, triclinic, space group *P*-1 (No. 2); *a* = 7.9657(5) Å, *b* = 10.7068(5) Å, *c* = 13.7205(8) Å, α = 91.683(4)°, β = 104.718(5)°, γ = 93.136(5)°, *V* = 1128.96(11) Å^3^; *Z* = 2; ρ = 1.684 g cm^−3^, μ(Mo K_α_) = 1.469 mm^−1^; θ_max_ = 28.000°, 20218 reflections measured, 5425 unique reflections and 3995 with *I* ≥ 2σ(*I*), *R*_int_ = 0.0346; Final *R* indices [(*I* > 2σ(*I*)]: *R* = 0.0411, *wR* = 0.1027, [all data]: *R* = 0.0614, *wR* = 0.1133, *S* = 1.123 for 334 parameters and 36 restraints, largest diff. peak and hole 0.511/−0.658 *e* Å^−3^.

Crystal data for **8**: 0.386 × 0.194 × 0.131 mm^3^; C_25_H_17_F_3_N_4_O, *M*_r_ = 446.42, triclinic, space group *P*-1 (No. 2); *a* = 8.2427(3) Å, *b* = 10.1166(4)Å, *c* = 13.1179(6) Å, α = 78.396(3)°, β = 78.370(3)°, γ = 83.739(3)°, *V* = 1046.84(8) Å^3^; *Z* = 2; ρ = 1.416 g cm^−3^, μ(Cu K*_α_*) = 0.907 mm^−1^; θ_max_ = 69.999°, 9006 reflections measured, 3939 unique reflections and 3494 with *I* ≥ 2σ(*I*), *R*_int_ = 0.0288; Final *R* indices [(*I* > 2σ(*I*)]: *R* = 0.0595, *wR* = 0.0641, [all data]: *R* = 0.1664, *wR* = 0.1727, *S* = 1.320 for 325 parameters and 35 restraints, largest diff. peak and hole 0.426/−0.307 *e* Å^−3^. For detailed description of crystal structures for compounds **5**–**8** please check Supporting Information File 1, Figures S22–S28 and Tables S2–S4.

### General procedure for the conventional click reactions of 1,2,3-triazole–quinoline derivatives **5–8**

Method 1a: Compound **4** (80 mg, 0.24 mmol) and the corresponding aryl azide (0.49 mL, 0.24 mmol) were dissolved in methanol (8 mL) and Cu(OAc)_2_ (2.24 mg, 0.05 equiv) was added. The reaction mixture was stirred for 3.5 h at 60 °C. The solvent was removed under reduced pressure and residue was purified by column chromatography on silica gel and Al_2_O_3_ with dichloromethane as eluent. We used here dichloromethane as an eluent as it is commonly used in similar systems, but it was shown that other mixtures, such as *n*-hexane/ethyl acetate (50:1) could also be efficient for the purification purposes. ESR spectroscopy showed no traces of copper in the purified product.

Method 1a*: Procedure as described in method 1a using compound **4** (1 equiv), the corresponding aryl azide (1 equiv) and Cu(OAc)_2_ (0.05 equiv) in methanol. The reaction mixture was stirred overnight at 60 °C.

Method 1b: To a mixture of CuI (1 mg, 4.9 mmol, 0.02 equiv), DIPEA (4.3 µL, 0.1 equiv) and HOAc (1.5 µL, 0.1 equiv) in dichlorometane (1.0 mL) 6-phenyl-4-(prop-2-ynyloxy)-2-(trifluoromethyl)quinoline (**4**, 80 mg, 0.24 mmol) and the corresponding azide (0.49 mL, 0.24 mmol) were added at room temperature. The reaction mixture was stirred for 3.5 h. The solvent was removed under reduced pressure and the residue was purified by column chromatography on silica gel and Al_2_O_3_ with dichloromethane as eluent.

### General procedure for the mechanochemical click reactions of 1,2,3-triazole–quinoline derivatives **5–8**

Method 2a: Compound **4** (80 mg, 0.24 mmol) and the corresponding aryl azide (0.49 mL, 0.24 mmol) were weighed in one half of the reaction vessel and the other half was filled with Cu(OAc)_2_ (2.24 mg, 0.05 equiv) and two 7 mm diameter stainless steel balls. The aryl azide solution was evaporated to dryness under vacuo, and the closed vessel was positioned in the IST500 mill. The mixture was ground for 3.5 h at 30 Hz and then purified by column chromatography on silica gel and Al_2_O_3_ with dichloromethane as eluent.

Method 2b: In one half of the reaction vessel we weighed azide (0.49 mL, 0.24 mmol), DIPEA (4.3 µL, 0.1 equiv) and acetic acid (1.5 µL, 0.1 equiv); the other half was filled with compound **4** (80 mg, 0.24 mmol) and CuI (1 mg, 4.9 mmol, 0.02 equiv), and two 7 mm diameter stainless steel balls (ball weight 1.3 g). The aryl azide solution was evaporated to dryness under vacuo, and the vessel was sealed and positioned in IST500 mill. The mixture was ground for 3.5 h at 30 Hz and then purified by column chromatography on silica gel and Al_2_O_3_ with dichloromethane as eluent.

Method 2c: In one half of the reaction vessel were weighed azide (0.49 mL, 0.24 mmol), DIPEA (4.3 µL, 0.1 equiv) and acetic acid (1.5 µL, 0.1 equiv) the other half was filled with compound **4** (80 mg, 0.24 mmol) and two brass balls each weighing 1.1 g. The aryl azide solution was evaporated to dryness under vacuo, and the vessel was sealed and positioned in IST500 mill. The mixture was ground for 3.5 h at 30 Hz and then purified by column chromatography on silica gel and Al_2_O_3_ with dichloromethane as eluent.

## Supporting Information

File 1 Solution synthetic procedures, characterization data, ^1^H, ^13^C NMR spectra of **4**–**8**, NOESY spectrum of **4**, high-resolution mass spectra of **5**–**8**, crystallographic data, FTIR–ATR, and Raman data.
