# Supplementary material for: Solvent-free copper-catalyzed click chemistry for the synthesis of N-heterocyclic hybrids based on quinoline and 1,2,3-triazole
Source: Beilstein J Org Chem. 2017 Nov 6;13:2352–63. doi: 10.3762/bjoc.13.232 (PMC5687011; doi:10.3762/bjoc.13.232)
Supplement: File 1 — Solution synthetic procedures, characterization data, 1H, 13C NMR spectra of 4–8, NOESY spectrum of 4, high-resolution mass spectra of 5–8, crystallographic data, FTIR–ATR, and Raman data. [file Beilstein_J_Org_Chem-13-2352-s001.pdf]

Supporting Information

for

Solvent-free copper-catalyzed click chemistry for the synthesis of *N*-heterocyclic hybrids based on quinoline and 1,2,3-triazole

Martina Tireli[‡,1] Silvija Maračić[‡,2] Stipe Lukin[1], Marina Juribašić Kulcsár[1], Dijana Žilić[1], Mario Cetina[3], Ivan Halasz[1], Silvana Raić-Malić[*,2] and Krunoslav Užarević[*,1]

Address: [1]Laboratory for Green Synthesis, Ruđer Bošković Institute, Bijenička 54, HR-10000 Zagreb, Croatia, [2]Department of Organic Chemistry, Faculty of Chemical Engineering and Technology, University of Zagreb, Marulićev trg 20, HR-10000 Zagreb, Croatia and [3]University of Zagreb, Faculty of Textile Technology, Department of Applied Chemistry, Prilaz baruna Filipovića 28a, HR-10000 Zagreb, Croatia

Email: Krunoslav Užarević - krunoslav.uzarevic@irb.hr; Silvana Raić-Malić - sraic@fkit.hr

*Corresponding author

‡Equally contributed to this work.

Solution synthetic procedures, characterization data, ^{1}H, ^{13}C NMR spectra of 4–8, NOESY spectrum of 4, high-resolution mass spectra of 5–8, crystallographic data, FTIR–ATR, and Raman data.

Table of contents

1. Solution procedures S2
2. ^{1}H and ^{13}C spectra of compounds **4–8** S6
3. HRMS spectra of compounds **5–8** S11
4. 2D NMR (NOESY) spectrum of compound **4** S15
5. FTIR–ATR spectra of compounds **5–8** S16
6. Raman data S18
7. X-ray crystal structure analysis S24

1. Solution procedures

*6-Phenyl-2-(trifluoromethyl)quinolin-4(1H)-one (**3**)*

A mixture of ethyl 4,4,4-trifluoro-3-oxobutanoate (**2**) (1.8 mL; mmol) and polyphosphoric acid (9.5 g) was heated at 100 °C and 4-aminobiphenyl (**1**, 2.0 g; 11.82 mmol) was added. The temperature was then raised at 150 °C and reaction mixture was stirred for 3 h. After cooling, the mixture was diluted with aqueous sodium hydroxide (50 mL) and the precipitate was then dissolved in a 10% aqueous sodium hydroxide (10 mL). After filtrating some insoluble material, clear solution was acidified with concentrated hydrochloric acid. The obtained solid was collected and crystallized from methanol. Compound **3** was isolated as white powder (1.54 g; 45 %; m.p. > 250 °C). ^{1}H-NMR (300 MHz, DMSO) (δ/ppm) 8.44 (s, 1H, H-5), 8.12 (dd, *J* = 31.0, 8.7 Hz, 2H, H-7, H-8), 7.83 (d, *J* = 7.6 Hz, 2H, Ph), 7.42−7.57 (m, 3H, Ph), 7.08 (s, 1H, H-3). ^{13}C-NMR (75 MHz, DMSO) (δ/ppm): 166.2 (C-4), 146.8 (C-2, q, *J* = 33.7 Hz), 145.2 (C-8a), 139.5 (C-6), 138.7 (C-1'), 131.0 (C-7), 129.6 (C-3',5'), 129.0 (C-8), 128.5 (C-4'), 127.5 (C-2',6'), 121.9 (CF_3, q, *J* = 275.2 Hz), 122.9 (C-4a), 120.2 (C-5), 101.8 (C-3).

*6-phenyl-4-(prop-2-yn-1-yloxy)-2-(trifluoromethyl)quinoline (**4**)*

To a suspension of 6-phenyl-2-(trifluoromethyl)quinolin-4(1*H*)-one (**3**, 700 mg; 2.42 mmol) in DMF (15 mL) K_2CO_3 (401 mg; 2.9 mmol) was added. After 30 min, propargyl bromide (0.32 mL; 2.42 mmol) was added and reaction mixture was stirred overnight. Solvent was removed under reduced pressure and crude residue was purified by column chromatography with dichloromethane as eluens. Compound **4** was isolated as off yellow solid (700 mg; 88%; m.p. = 113–115 °C). ^{1}H-NMR (300 MHz, DMSO) (δ/ppm) 8.39 (d, *J* = 1.5 Hz, 1H, H-5), 8.17–8.27 (m, 2H, H-7, H-8), 7.81–7.88 (m, 2H, Ph), 7.42–7.60 (m, 4H, Ph, H-3), 5.34 (d, *J* = 2.4 Hz, 2H, CH_2), 3.80 (t, *J* = 2.3 Hz, 1H, CH). ^{13}C-NMR (75 MHz, DMSO) (δ/ppm) 162.1 (C-4), 148.0 (C-2, q, *J* = 33.7 Hz), 147.3 (C-8a), 140.3 (C-6), 139.3 (C-1'), 131.2 (C-7), 130.4

(C-8), 129.7 (C-3',5'), 128.8 (C-4'), 127.7 (C-2',6'), 122.0 (CF_3, q, J = 276.0 Hz), 121.9 (C-4a),119.1 (C-5), 99.2 (C-3), 80.4 (C-2''), 78.1 (C-3''), 57.7 (C-1'').

4-{[1-(4-chlorophenyl)-1H-1,2,3-triazol-4-yl]methoxy}-6-phenyl-2-(trifluoromethyl)quinoline ***(5)***

Compound **5** was isolated as colourless crystals (m.p. = 225–227 °C) by method 2a (67 mg; 57%); method 2b (100 mg; 85%); method 2c (90 mg; 77%); method 1a (24 mg; 21%); method 1a* (89 mg; 77%) and method 1b (7 mg; 5%). ^{1}H-NMR (300 MHz, DMSO) (δ/ppm): 9.12 (s, 1H, H-triazole), 8.38 (s, 1H, H-5), 8.15–8.26 (m, 2H, H-7, H-8), 7.96–8.01 (m, 2H, Ph), 7.77–7.81 (m, 3H, Ph, H-3), 7.67–7.72 (m, 2H, Ph), 7.41–7.55 (m, 3H, Ph), 5.76 (s, 2H, CH_2). ^{13}C-NMR (151 MHz, DMSO) (δ/ppm): 162.4 (C-4), 147.8 (q, J = 33.7 Hz), 146.8 (C-8a), 142.8 (C-triazole), 139.8 (C-6), 138.9 (C-1'), 135.3 (C-1''), 133.2 (C-4''), 130.7 (C-7), 129.9 (C-8,C-3',5'), 129.2 (C-3'',5''), 128.2 (C-4'), 127.3 (C-2',6'), 121.5 (CF_3, q, J = 275.6 Hz), 123.6 (CH-triazole), 122.0 (C-2'',6''), 121.4 (C-4a), 118.8 (C-5), 98.7 (C-3), 62.6 (CH_2). HRMS (ESI): calcd. for $C_{25}H_{16}ClF_3N_4O$ $[M+H]^+$ = 481.1043; found = 481.1046.

4-{[1-(4-bromophenyl)-1H-1,2,3-triazol-4-yl]methoxy}-6-phenyl-2-(trifluoromethyl)quinoline ***(6)***

Compound **6** was isolated as colourless crystals (m.p. = 239–241 °C) by method 2a (77 mg; 60%); method 2b (112 mg; 87%); method 2c (103 mg; 80%); method 1a (58 mg; 45%); method 1a* (96 mg; 76%) and method 1b (52 mg; 40%). ^{1}H-NMR (600 MHz, DMSO) (δ/ppm): 9.14 (s, 1H, H-triazole), 8.39 (d, J = 1.6 Hz, 1H, H-5), 8.20–8.24 (m, 2H, H-7, H-8), 7.93 (d, J = 8.8 Hz, 2H, Ph), 7.76–7.85 (m, 5H, Ph, H-3), 7.53 (t, J = 7.7 Hz, 2H, Ph), 7.45 (t, J = 7.4 Hz, 1H, Ph), 5.76 (d, J = 2.0 Hz, 2H, CH_2). ^{13}C-NMR (75 MHz, DMSO) (δ/ppm): 162.9 (C-4), 147.8 (C-2, J = 33.6 Hz), 147.3 (C-8a), 143.3 (C-triazole), 140.2 (C-6), 139.4 (C-1'), 136.2 (C-1''), 133.3 (C-3'',5''), 131.2 (C-7), 130.4 (C-8), 129.7 (C-3',5'), 128.7 (C-4'), 127.8 (C-2',6'), 124.0 (CH-triazole), 122.7 (C-2'',6''), 122.0 (C-4''), 121.9 (C-4a), 121.5 (CF_3,

q, J = 275.6 Hz), 119.3 (C-5), 99.2 (C-3), 63.2 (CH_2). HRMS (ESI): calcd. for $C_{25}H_{16}BrF_3N_4O$ $[M+H]^+$ = 525.0538; found = 525.0525.

4-{[1-(4-iodophenyl)-1H-1,2,3-triazol-4-yl]methoxy}-6-phenyl-2-(trifluoromethyl)quinoline (7)

Compound **7** was isolated as colourless crystals (m.p. = 254–256 °C) by method 2a (108 mg; 77%); method 2b (129 mg; 92%); method 2c (122 mg; 87%); method 1a (124 mg; 89%); method 1a* (124 mg; 89%) and method 1b (72 mg; 52%). ^{1}H-NMR (600 MHz, DMSO) (δ/ppm): 9.12 (s, 1H, H-triazole), 8.38 (d, J = 1.6 Hz, 1H, H-5), 8.18–8.25 (m, 2H, H-7, H-8), 7.98 (d, J = 8.7 Hz, 2H, Ph), 7.73–7.83 (m, 5H, Ph, H-3), 7.52 (t, J = 7.7 Hz, 2H, Ph), 7.44 (t, J = 7.3 Hz, 1H, Ph), 5.75 (s, 2H, CH_2). ^{13}C-NMR (151 MHz, DMSO) (δ/ppm): 162.4 (C-4), 147.8 (C-2, J = 33.6 Hz), 146.8 (C-8a), 142.8 (C-triazole), 139.7 (C-6), 138.9 (C-1'), 138.6 (C-3'',5''), 136.2 (C-1''), 130.7 (C-7), 129.9 (C-8), 129.2 (C-2'',6''), 128.2 (C-4'), 127.3 (C-3',5'), 121.5 (CF_3, q, J = 275.6 Hz), 123.4 (CH-triazole), 122.1 (C-2',6'), 121.4 (C-4a), 118.8 (C-5), 98.7 (C-3), 94.6 (C-4''), 62.7 (CH_2). HRMS (ESI): calcd. for $C_{25}H_{16}IF_3N_4O$ $[M+H]^+$ = 573.0399; found = 573.0389.

*6-phenyl-4-[(1-phenyl-1H-1,2,3-triazol-4-yl)methoxy]-2-(trifluoromethyl)quinoline (**8**)*

Compound **8** was isolated as colourless crystals (m.p. = 212–214 °C) by method 2a (78 mg; 72%); method 2b (86 mg; 79%); method 2c (83 mg; 76%); method 1a (11 mg; 10%); method 1a* (23 mg; 21%) and method 1b (5 mg; 5%). ^{1}H-NMR (600 MHz, DMSO) (δ/ppm): 9.10 (s, 1H, H-triazole), 8.39 (d, J = 1.7 Hz, 1H, H-5), 8.19–8.23 (m, 2H, H-7, H-8), 7.94 (d, J = 7.7 Hz, 2H, Ph), 7.80 (d, J = 7.3 Hz, 2H, Ph), 7.77 (s, 1H, H-3), 7.62 (t, J = 7.9 Hz, 2H, Ph), 7.50–7.53 (m, 3H, Ph), 7.44 (t, J = 7.4 Hz, 1H, Ph), 5.76 (s, 2H, CH_2). ^{13}C-NMR (151 MHz, DMSO) (δ/ppm): 162.4 (C-4), 147.8 (q, J = 33.7 Hz, C-2), 146.8 (C-8a), 142.6 (C-triazole), 139.7 (C-6), 138.9 (C-1'), 136.5 (C-1''), 130.7 (C-7), 129.9 (C-8, C-3',5'), 129.2 (C-3'',5''), 128.9 (C-4''), 128.2 (C-4'), 127.3 (C-2',6'), 121.5 (CF_3, q, J = 275.4 Hz), 123.5 (CH-triazole),

120.6 (C-4a), 120.3 (C-2'',6''), 118.8 (C5), 98.7 (C-3), 62.7 (CH_2). HRMS (ESI): calcd. for $C_{25}H_{17}F_3N_4O$ $[M+H]^+$ = 447.1433; found = 447.1448.

2. ^{1}H and ^{13}C spectra of compounds 4–8

a)

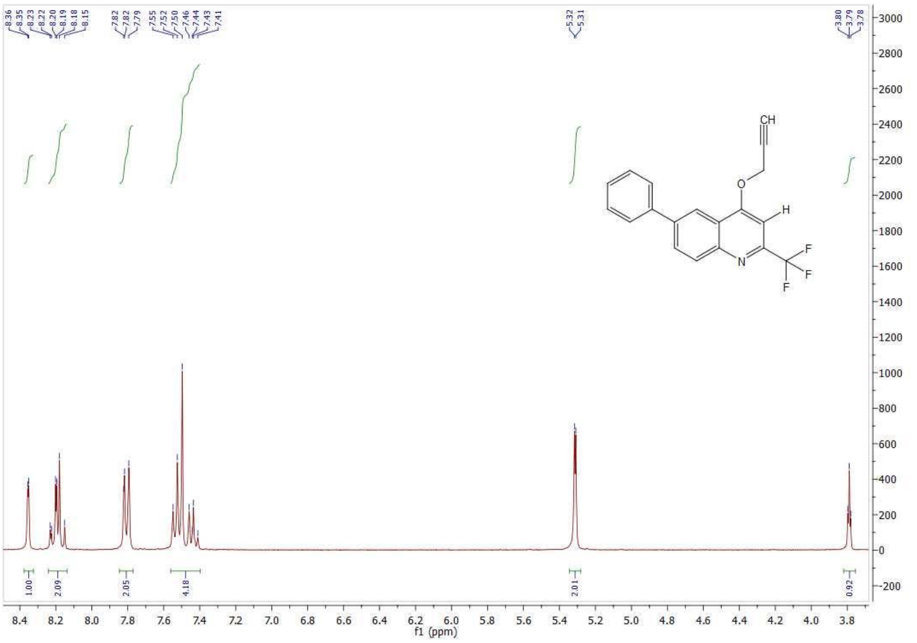

b)

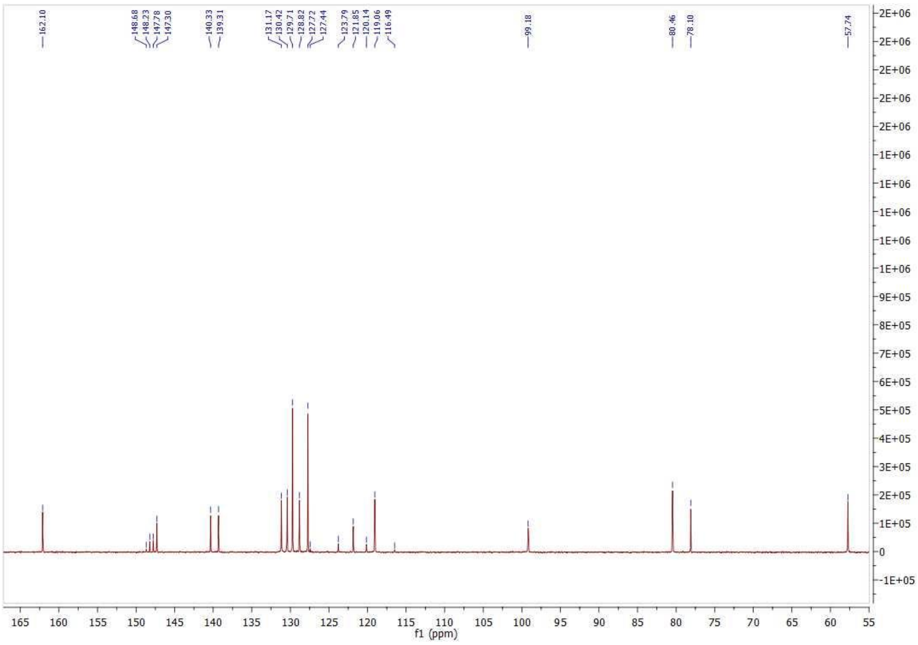

Figure S1. a) ^{1}H NMR and b) ^{13}C NMR of compd. **4**.

a)

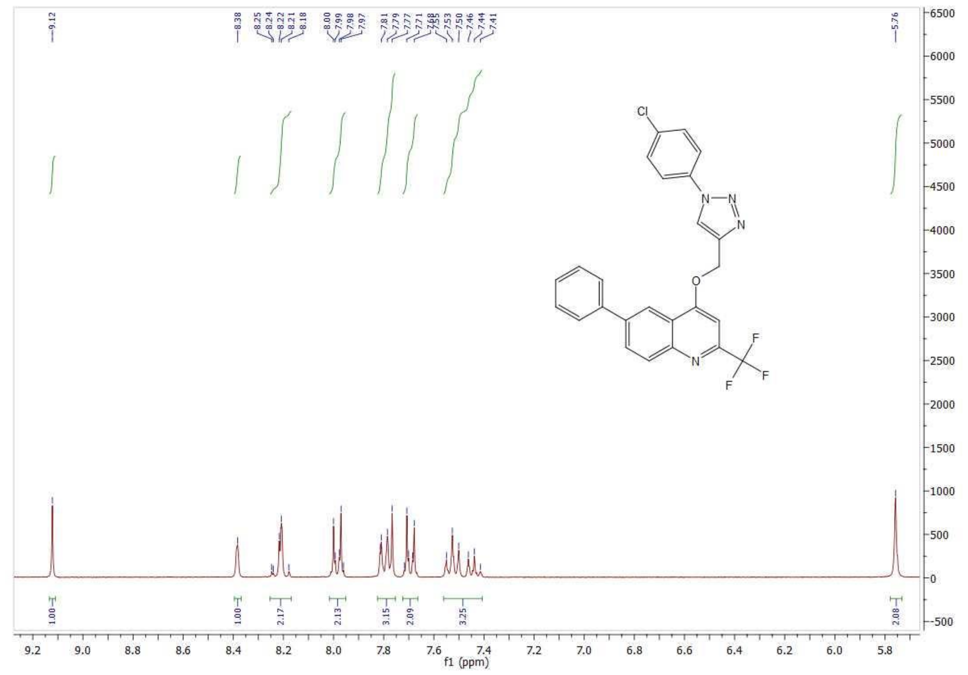

b)

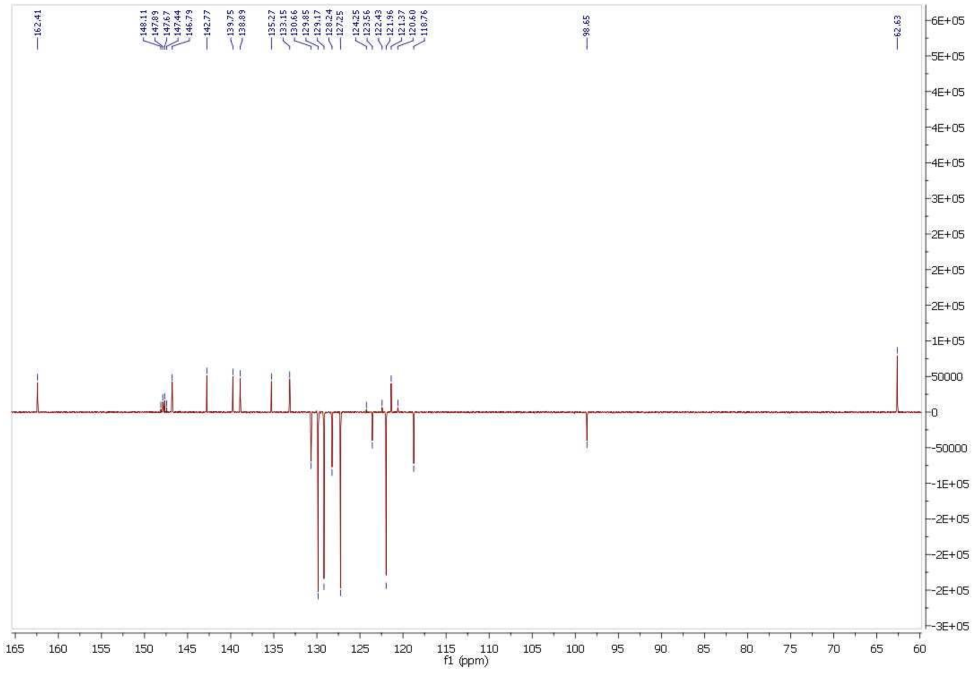

Figure S2. a) ^{1}H NMR and b) ^{13}C NMR of compd. **5**.

a)

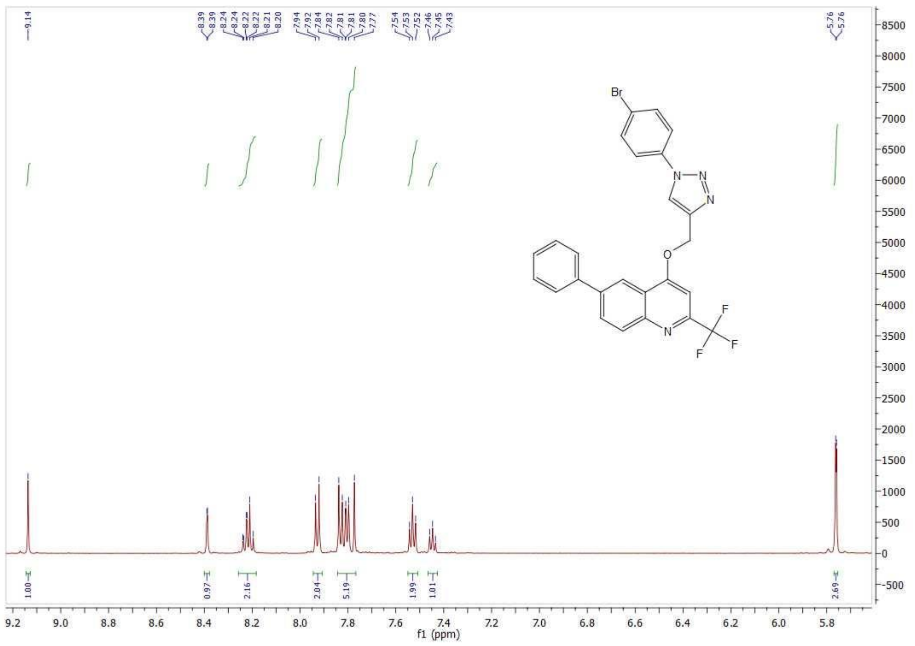

b)

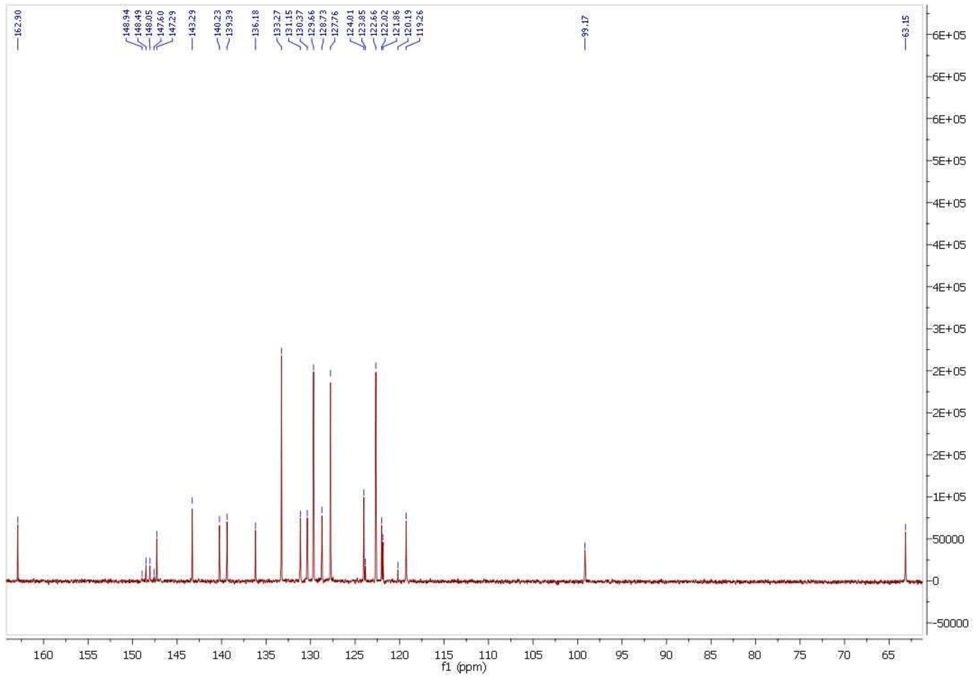

Figure S3. a) ^{1}H NMR and b) ^{13}C NMR of compd. **6**.

a)

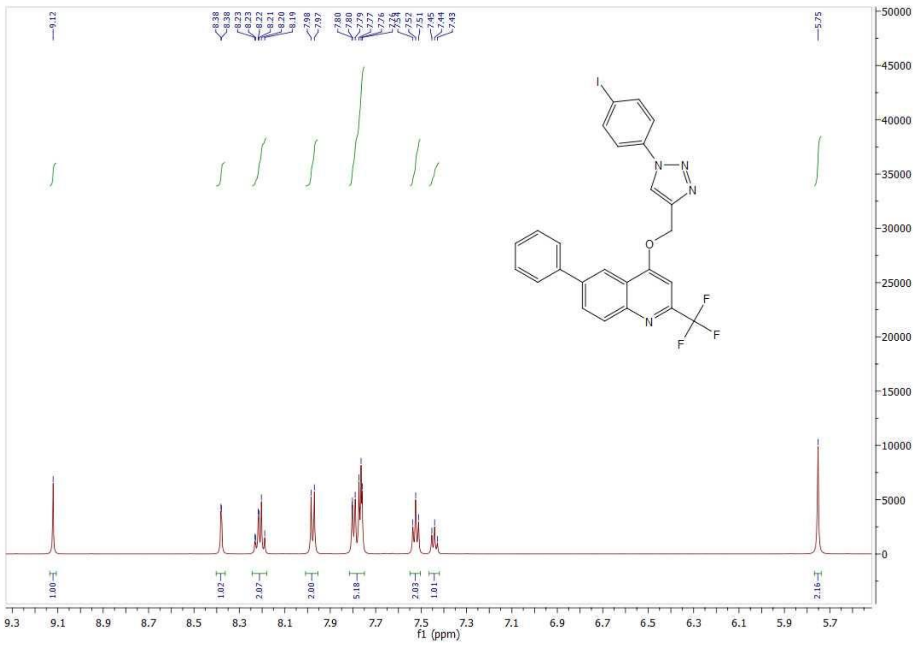

b)

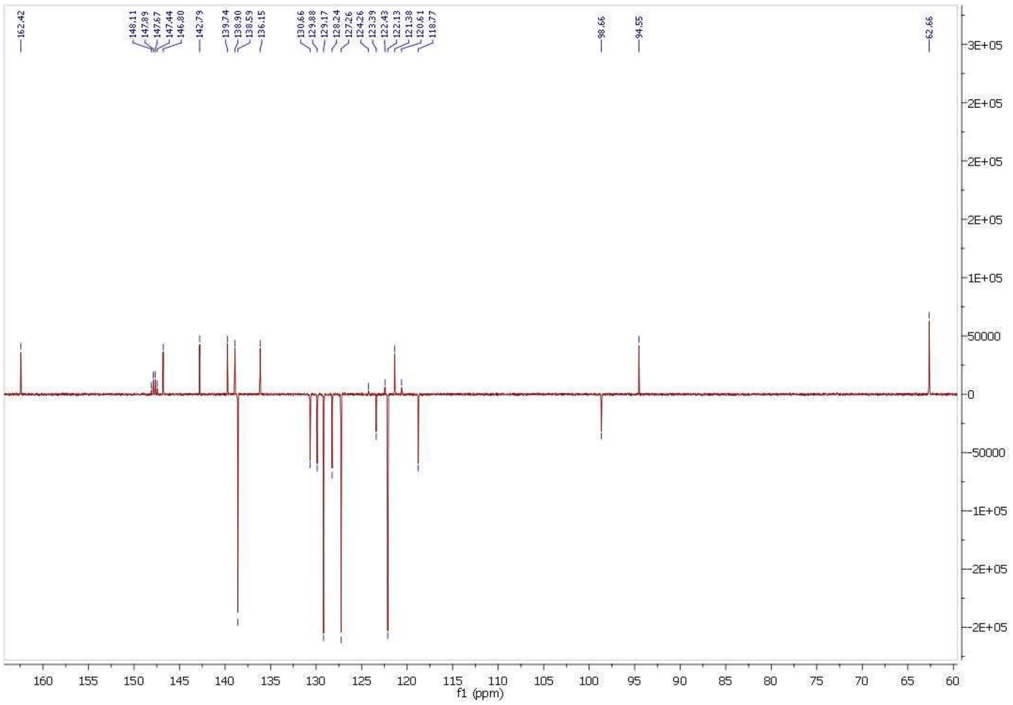

Figure S4. a) ^{1}H NMR and b) ^{13}C NMR of compd. **7**.

a)

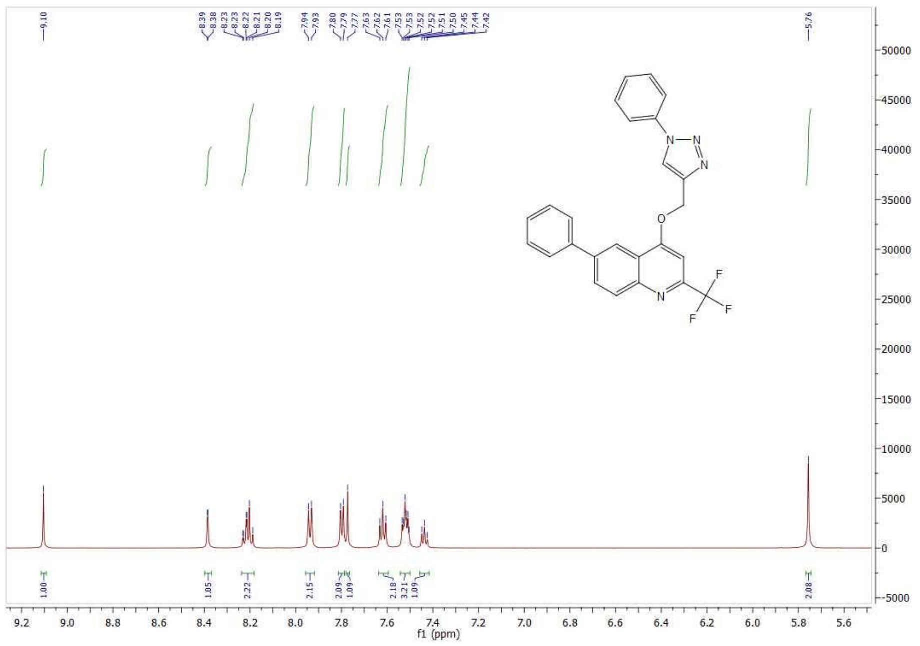

b)

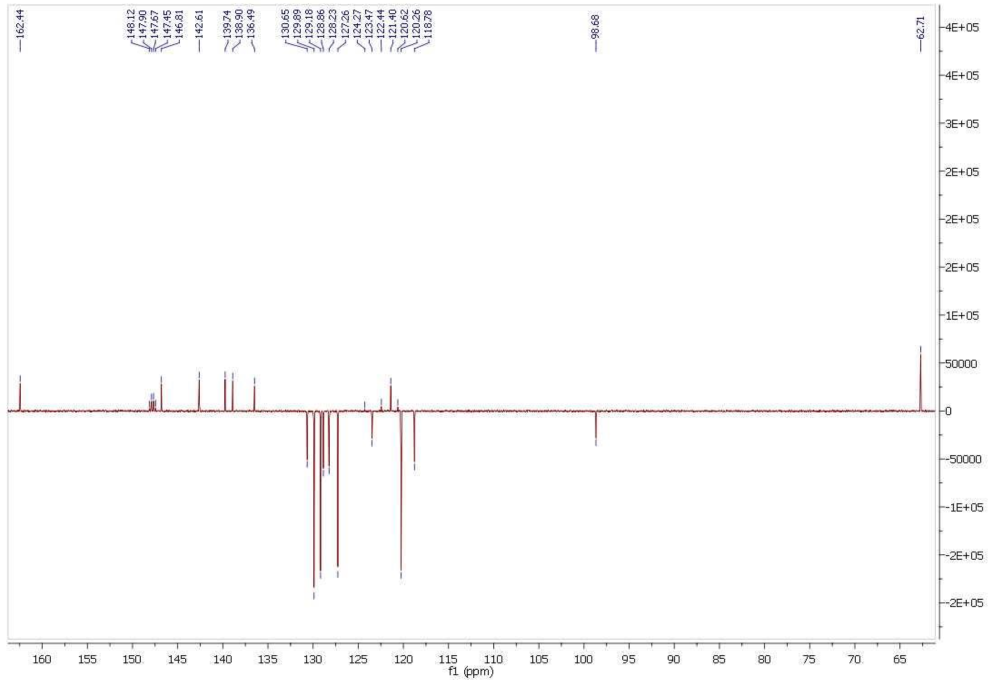

Figure S5. a) ^{1}H NMR and b) ^{13}C NMR of compd. **8**.

3. HRMS spectra of compounds 5–8

a)

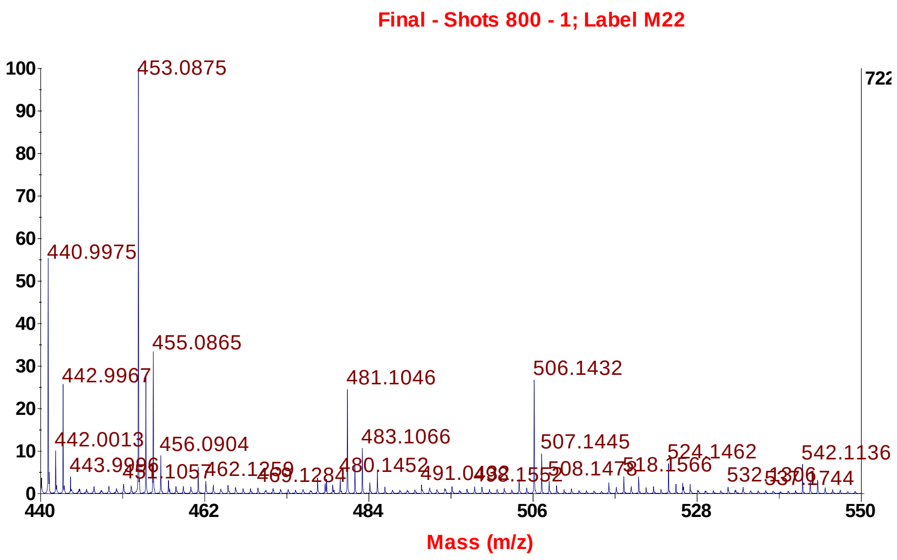

b)

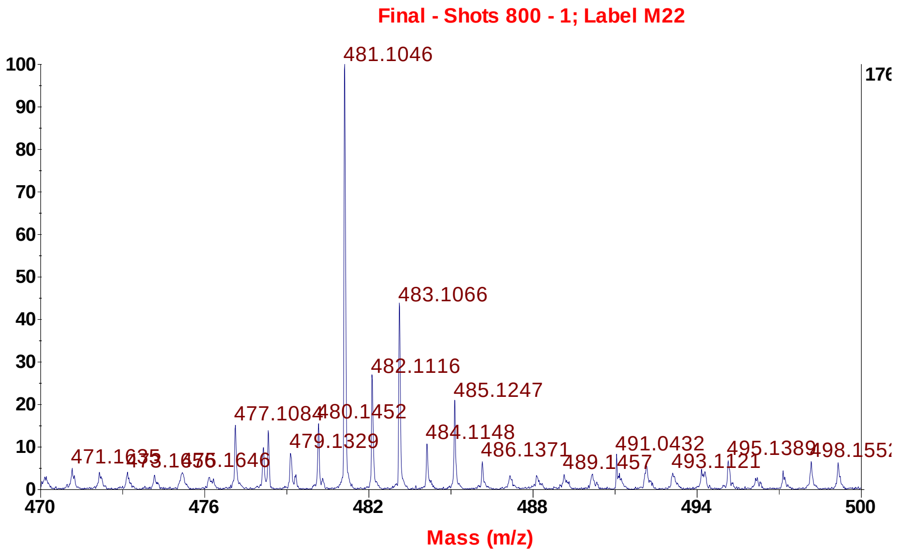

Figure S6. HRMS spectra of compd. **5**.

a)

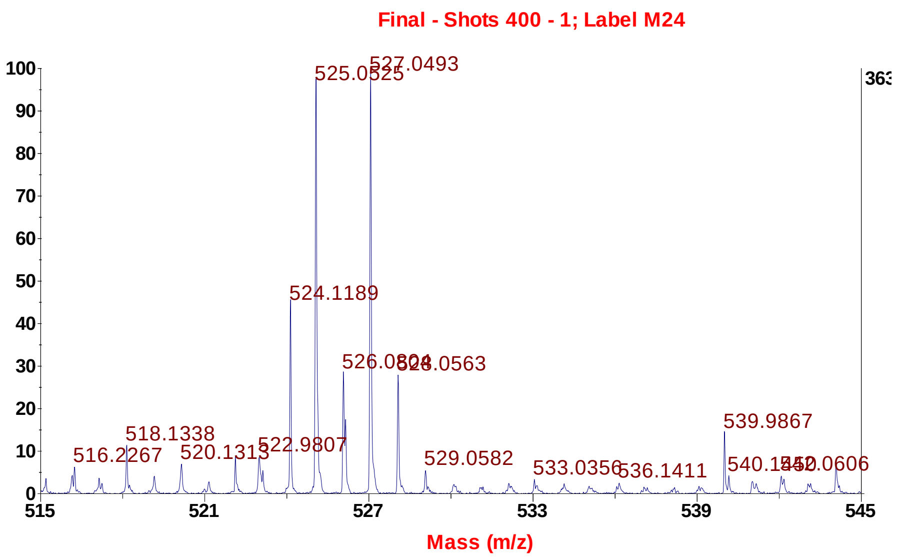

b)

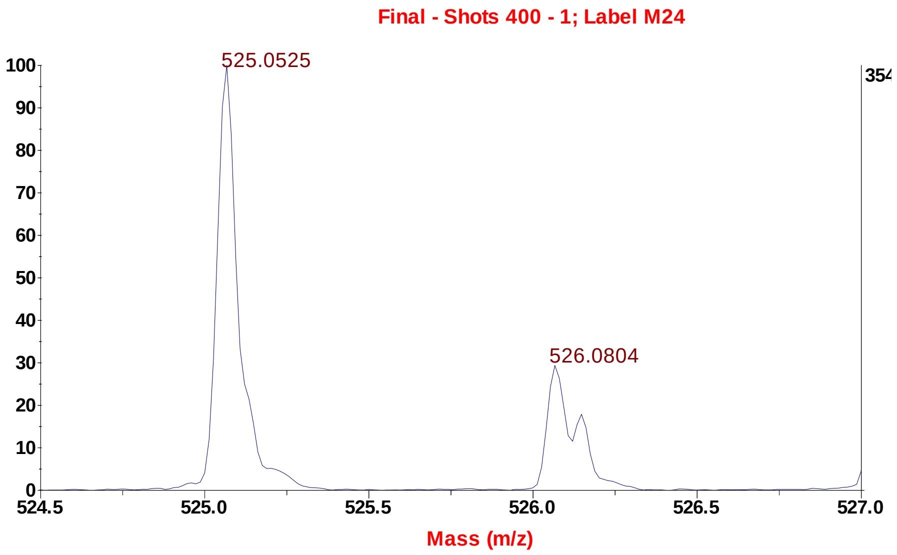

Figure S7. HRMS spectra of compd. **6**.

a)

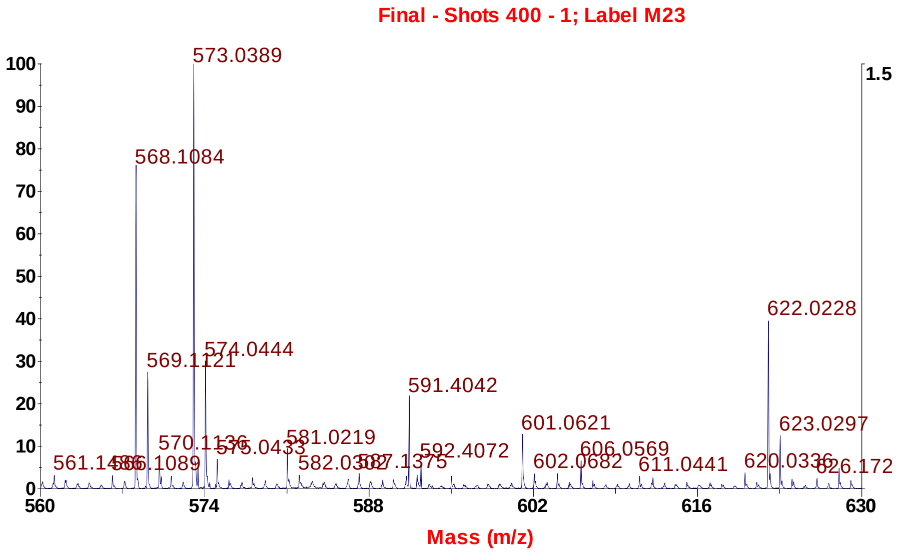

b)

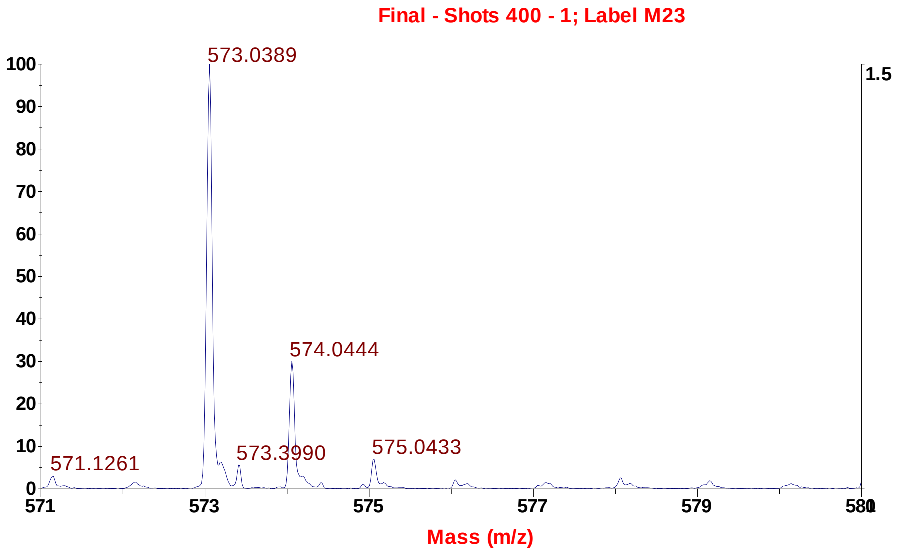

Figure S8. HRMS spectra of compd. **7**.

a)

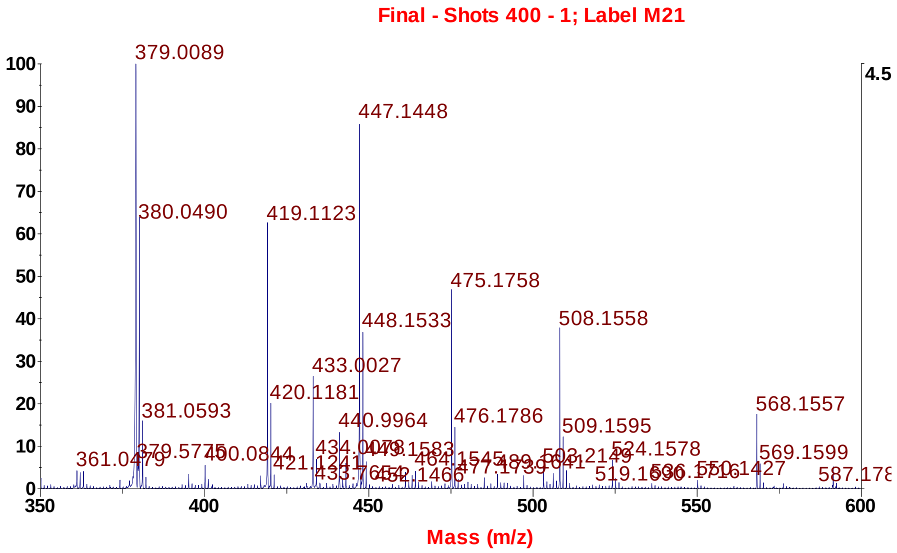

b)

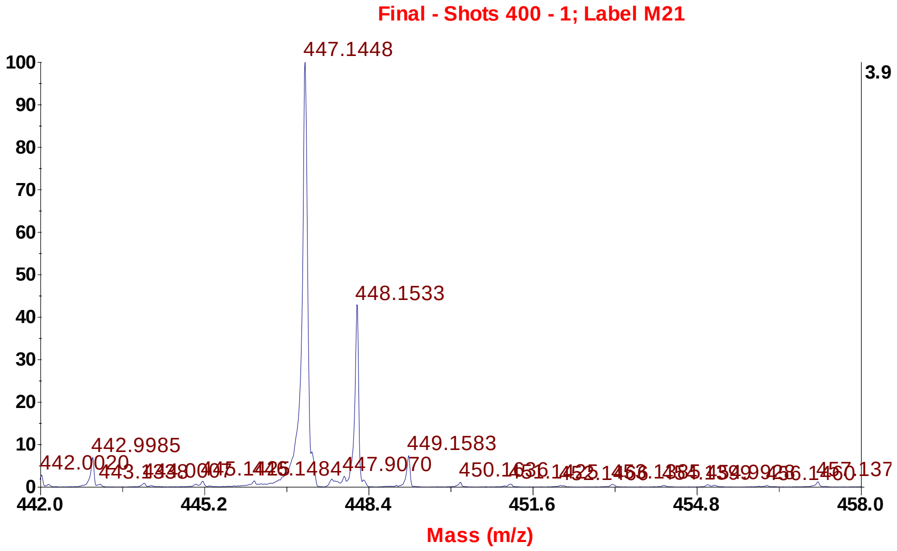

Figure S9. HRMS spectra of compd. **8**.

4. 2D NMR (NOESY) spectrum of compound 4

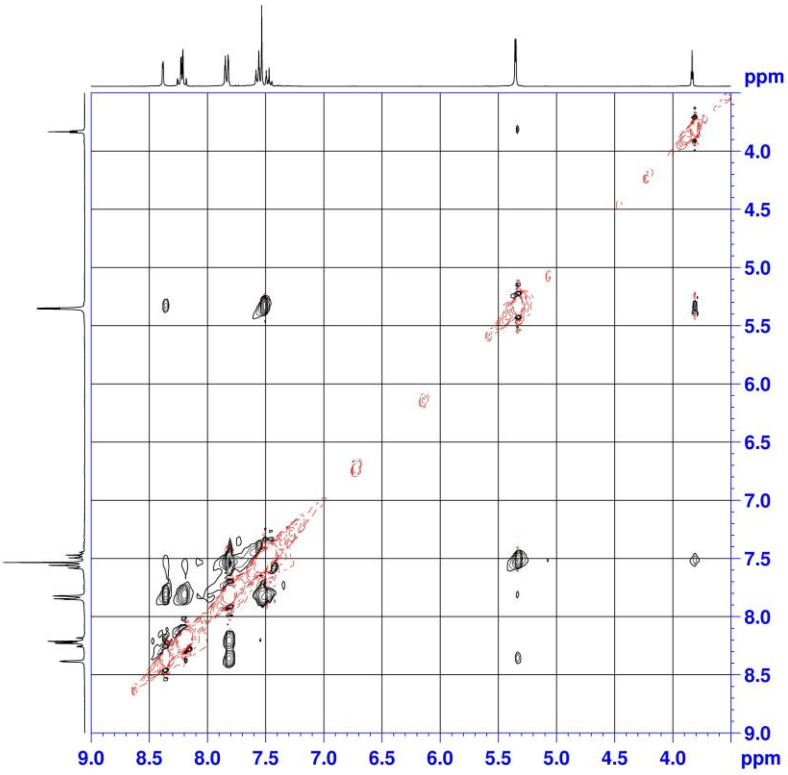

Figure S10. 2D NMR (NOESY) spectrum of compd. **4**.

5. FTIR–ATR spectra of compounds 5–8

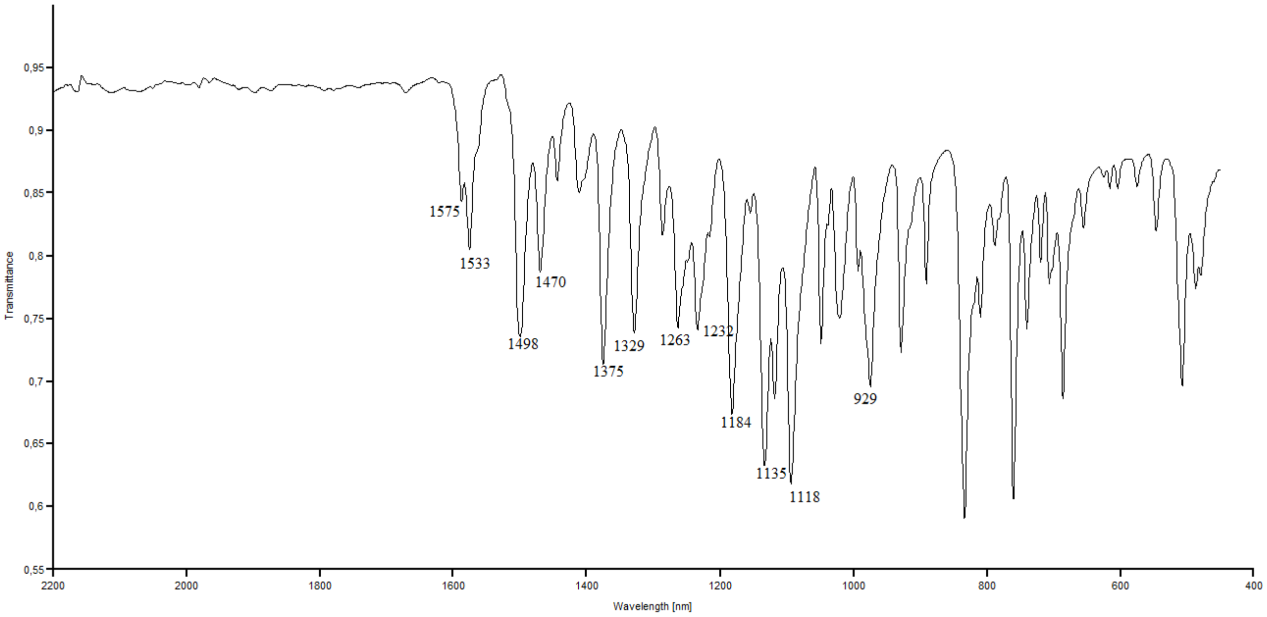

Figure S11. FTIR–ATR spectrum of **5**.

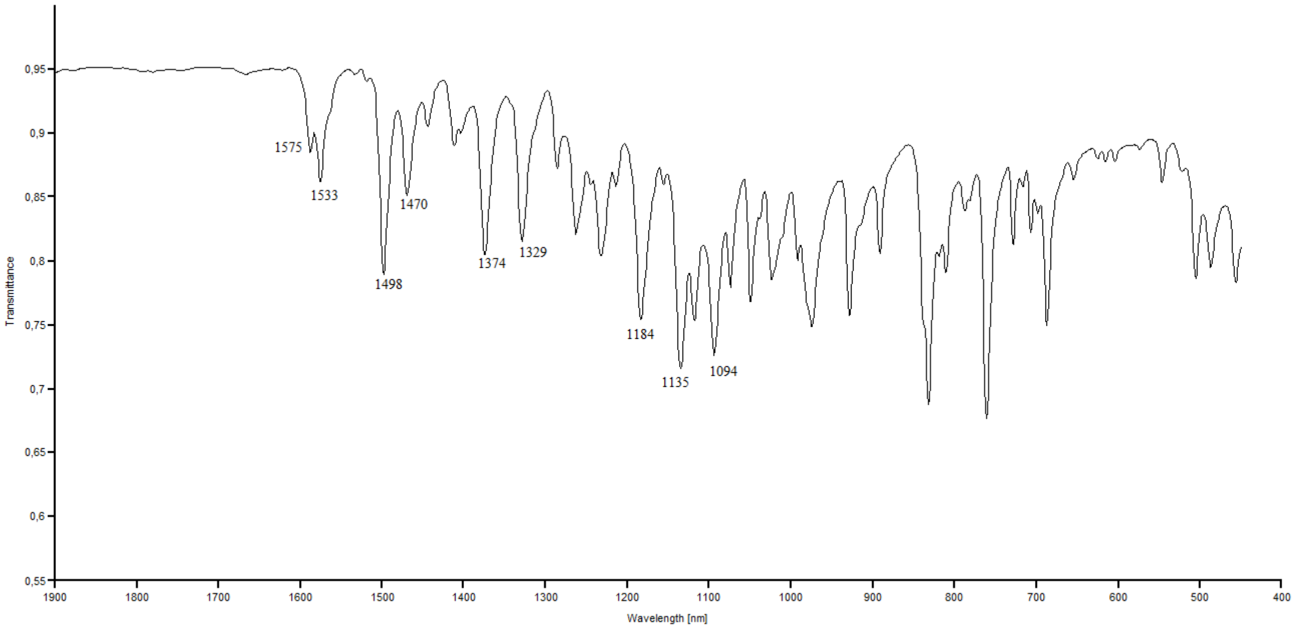

Figure S12. FTIR–ATR spectrum of **6**.

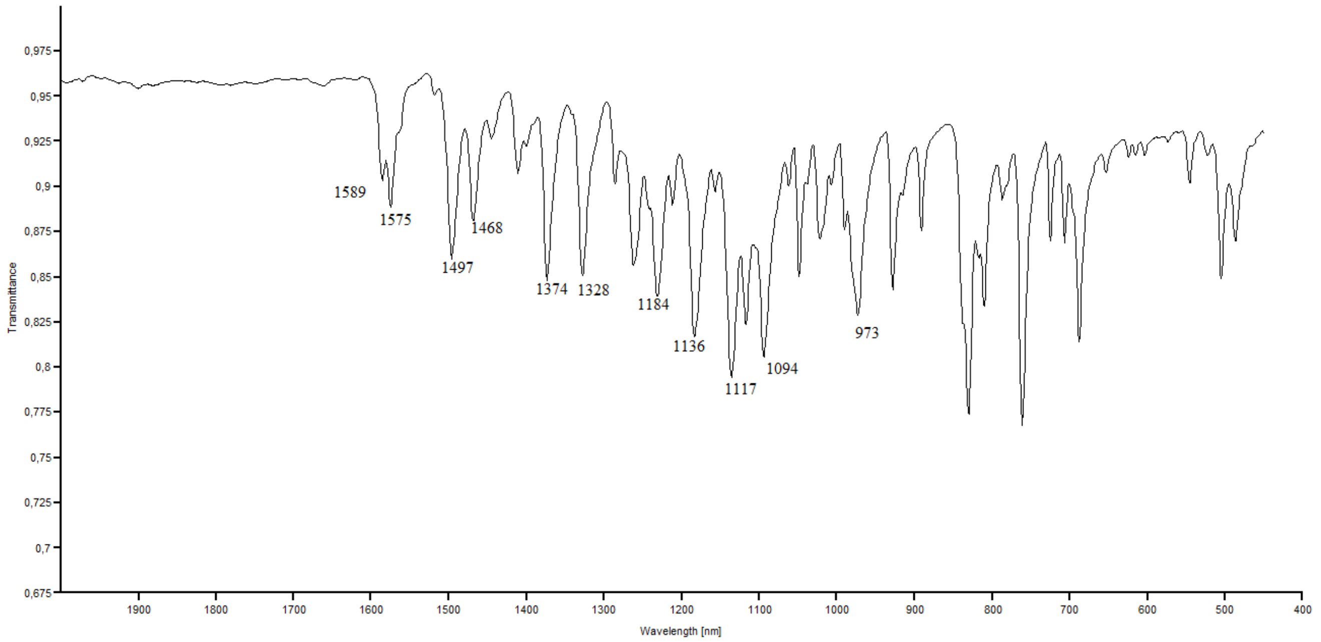

Figure S13. FTIR–ATR spectrum of **7**.

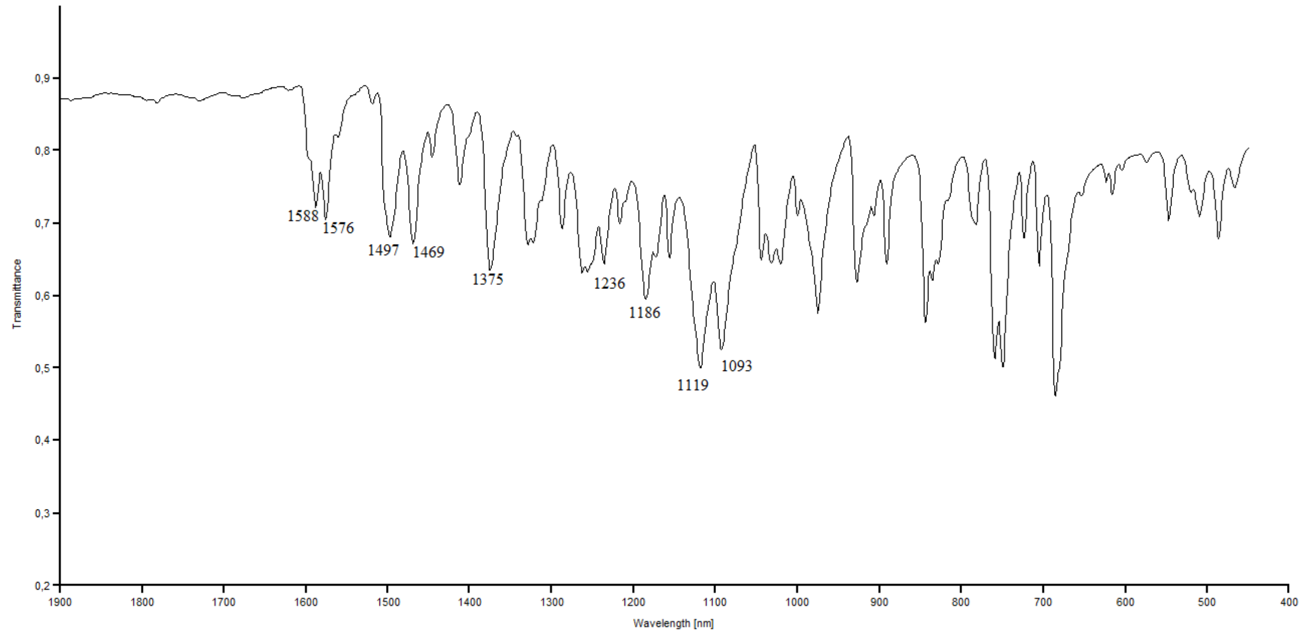

Figure S14. FTIR–ATR spectrum of **8**.

6. Raman data

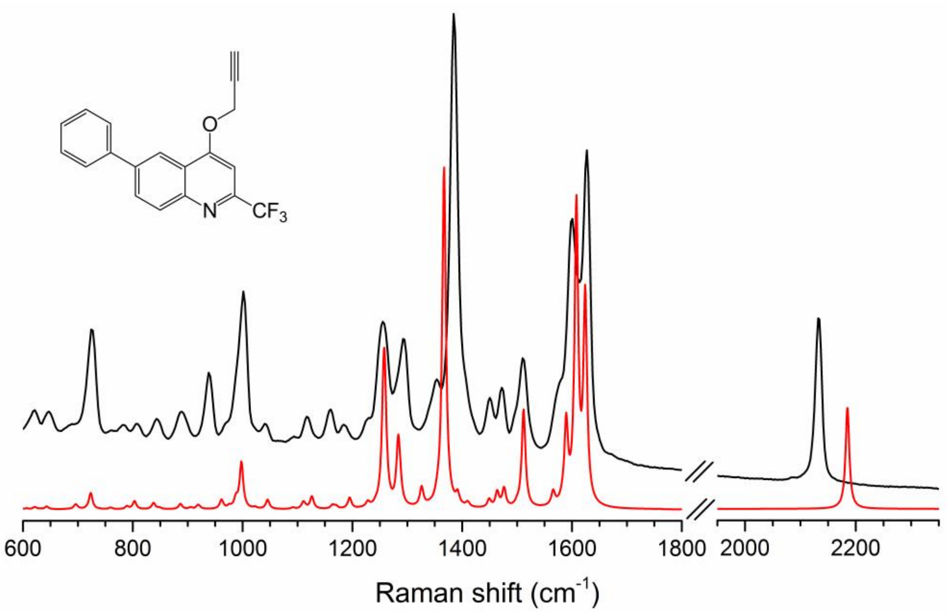

Figure S15. Experimental (**black**) and calculated (**red**) Raman spectrum of alkyne **4**.

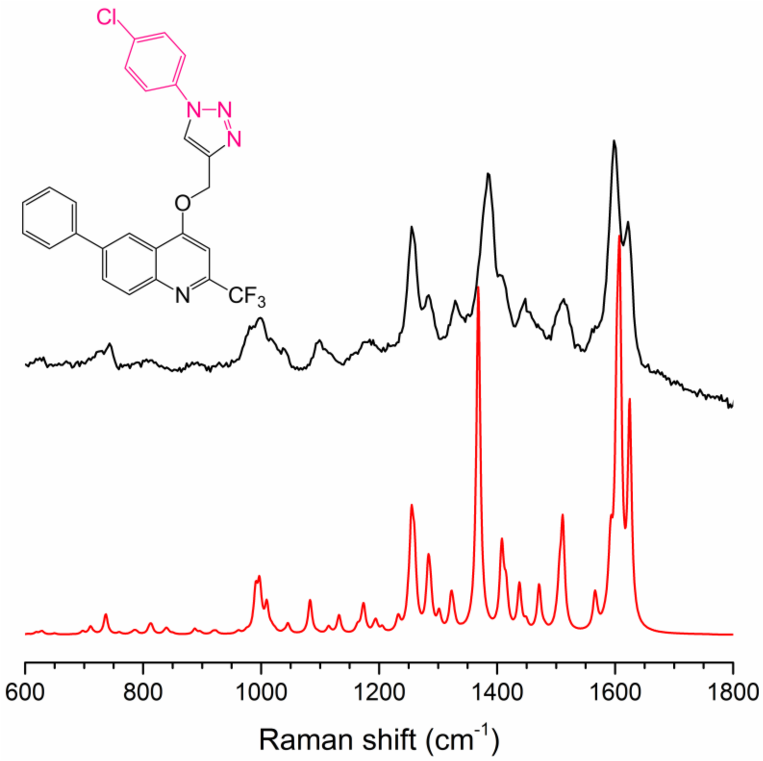

Figure S16. Experimental (**black**) and calculated (**red**) Raman spectrum of product **5** (R = Cl).

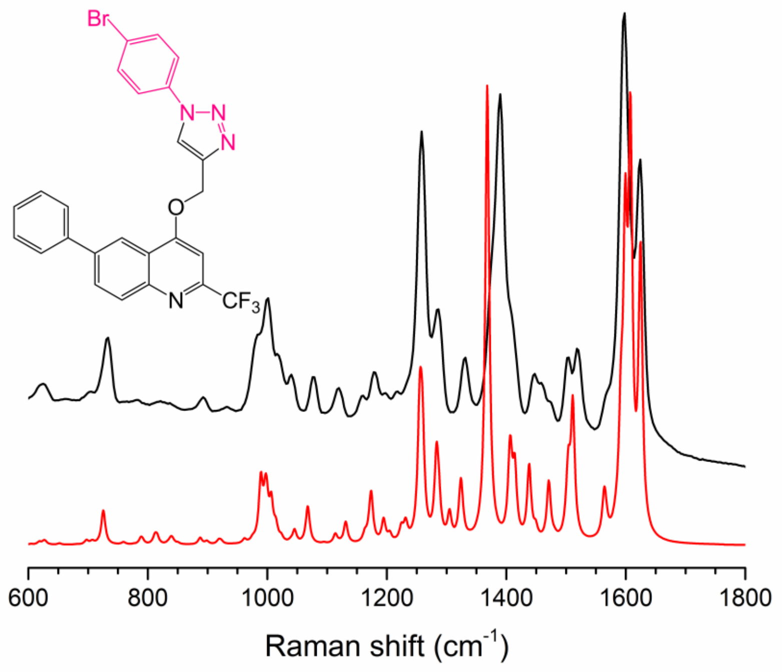

Figure S17. Experimental (**black**) and calculated (**red**) Raman spectrum of product **6** (R = Br).

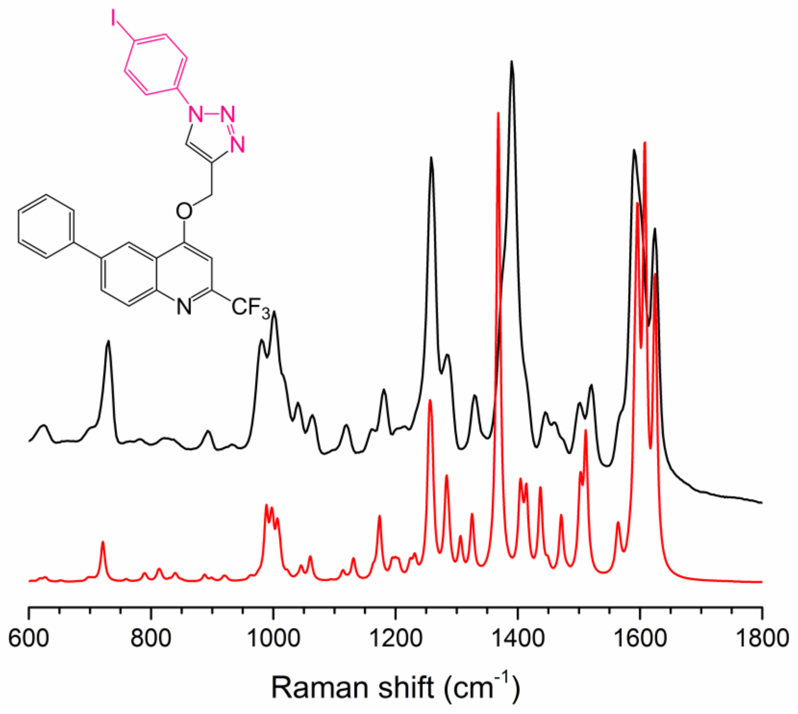

Figure S18. Experimental (**black**) and calculated (**red**) Raman spectrum of product **7** (R = I).

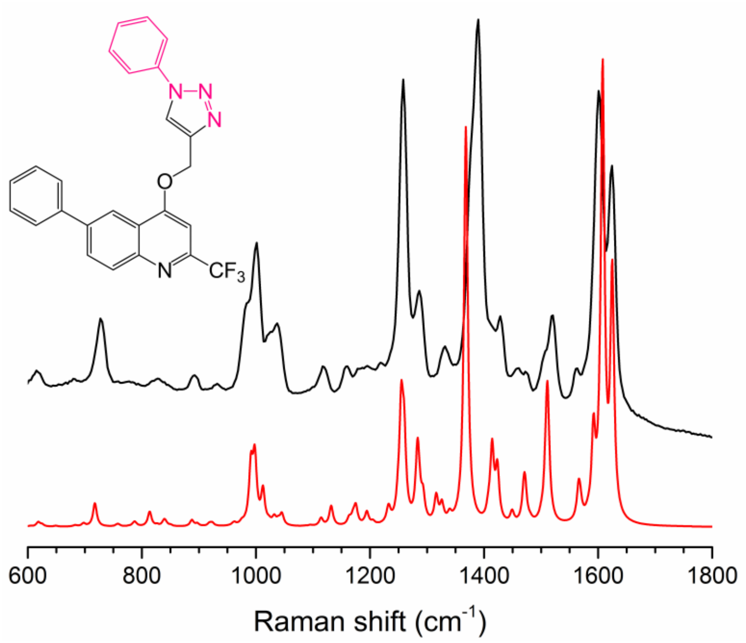

Figure S19. Experimental (**black**) and calculated (**red**) Raman spectrum of product **8** (R = H).

Table S1. Experimental shifts[1] (cm^{-1}) and proposed assignment[2] of selected Raman bands for compounds **4**–**8**. Bands marked with an asterisk are either too low in intensity or heavily overlapped.

**4**	**5**	**6**	**7**	**8**	Triazole[3]	Ph2[3,4]	Other aromatic groups[4]	Alkyl groups
2133 m	-	-	-	-	-	-	-	ν(C≡C)
1627 vs	1622 s	1624 s	1624 s	1624 s	-	-	ν(C=C) [Q2], β(CH) [Q2]	-
1600 s	1598 vs	1598 vs	1590 vs	1601 s	-	ν(C=C), β(CH)	ν(C=C) [Q1, Ph1], β(CH) [Q1, Ph1]	-
1510 w	1513 br w	1518 w	1521 w	1518 w	-	-	ν(C=C) [Q2, Ph1], β(CH) [Q2, Ph1]	-
-	1409 sh m	1407 sh	1410 sh w	*	ν(CN), β(CH)	ν(C=C), β(CH)	ν_{as}(CCN) [Q1], ν(CC) [Q2], β(CH) [all]	ω(OCH_2)
1384 vs	1387 vs	1390 vs	1390 vs	1390 vs	-	-	ν_{as}(CCC) [Q1-Q2], ν_{as}(CCN) [Q1-Q2], β(CH) [Q2]	-
-	1330 w	1331 w	1329 w	1330 w	ν_s(NNN), β(CH)	ν(C=C), β(CH)	ν(CN) [Q1], β(CH) [all]	ω(OCH_2)
1293 w	1284 w	1286 m	1285 w	1287 w	-	ν_{as}(CCC)	ν_{as}(CCC) [Ph1], δ_s(CCC) [Q2], ν_{as}(CCN) [Q1-Q2], β(CH) [Q1, Q2, Ph1]	-
1255 w	1255 s	1258 s	1258 vs	1258 s	ν_{as}(CN=N)	-	ν(CCN) [Q1-Q2], ν_{as}(C-CC) [Q2-Ph1], β(CH) [Q1, Q2, Ph1]	ν(C-CF_3), τ(OCH_2)
-	1099 br w	1077 w	1064 w	*	-	ν_s(CC_xC), β(CH)	-	-
1001 m	999 br w	1001 br m	1001 br w	1001 br m	ν(N-N), δ_s(CN-N), β(CH)	ν_s(CCC), β(CH)	ν_s(CCC) [Q2, Ph1], β(CH) [Q2], ring breathe [Ph1]	ν(O-CH_2), ρ(OCH_2)
938 w	-	-	-	-	-	-	γ(CH) [Ph1]	ν_s(O-CH_2-C)
726 w	*	730 m	730 w	729 w	-	δ_S(CCC)	δ_S(CCC) [Q2, Ph1]	ν_s(CF_3)

[1] Band descriptors: s – strong, m – medium, w – weak, v – very, sh – shoulder.
[2] Vibrational mode descriptors: ν - stretching, β - in-plane bending, γ - out-of-plane bending, ω - wagging, τ - twisting, ρ - rocking, s – symmetric, as – asymmetric.
[3] Only for triazole products.
[4] Q1 is a pyridyl ring and Q2 is a phenyl ring in a quinolinyl group. Phenyl rings originating from the alkyne and the azide are marked with Ph1 and Ph2, respectively.

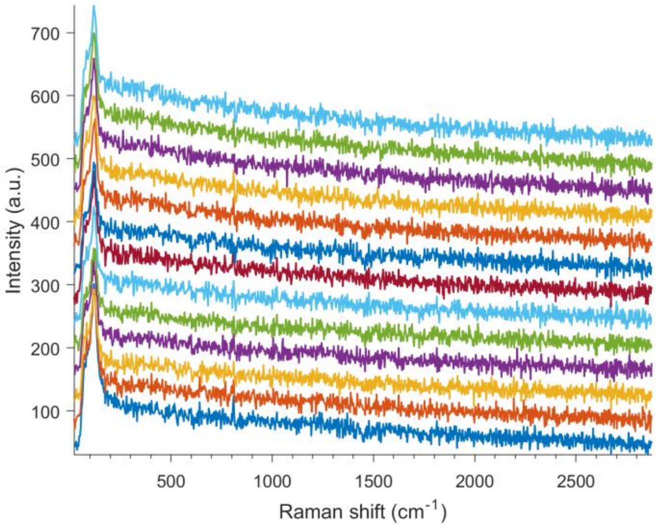

a)

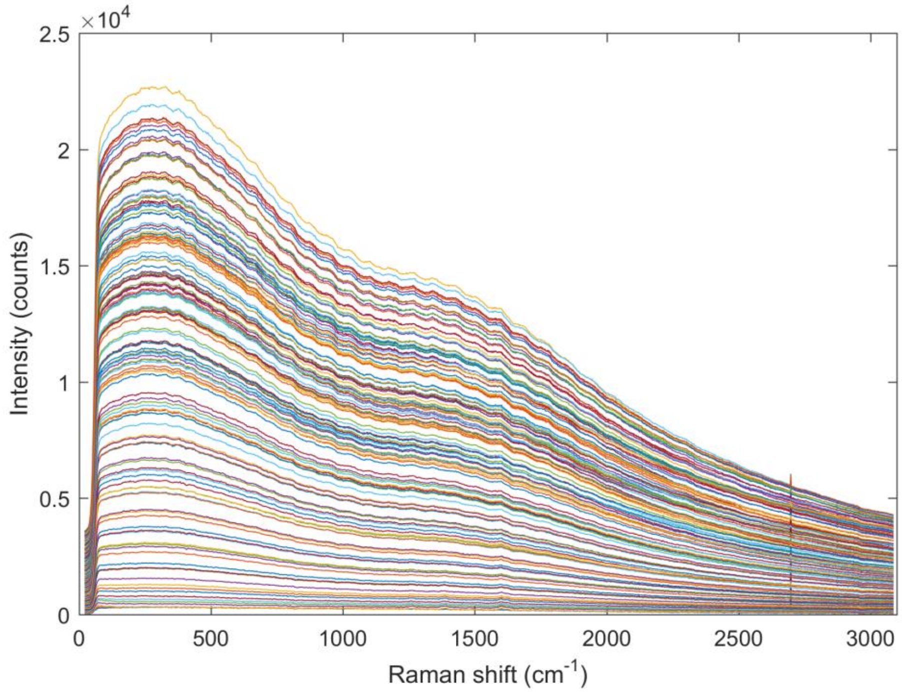

b)

Figure S20. In situ Raman monitoring of mechanochemical milling of a) CuI, and b) the mixture of product **5** and CuI.

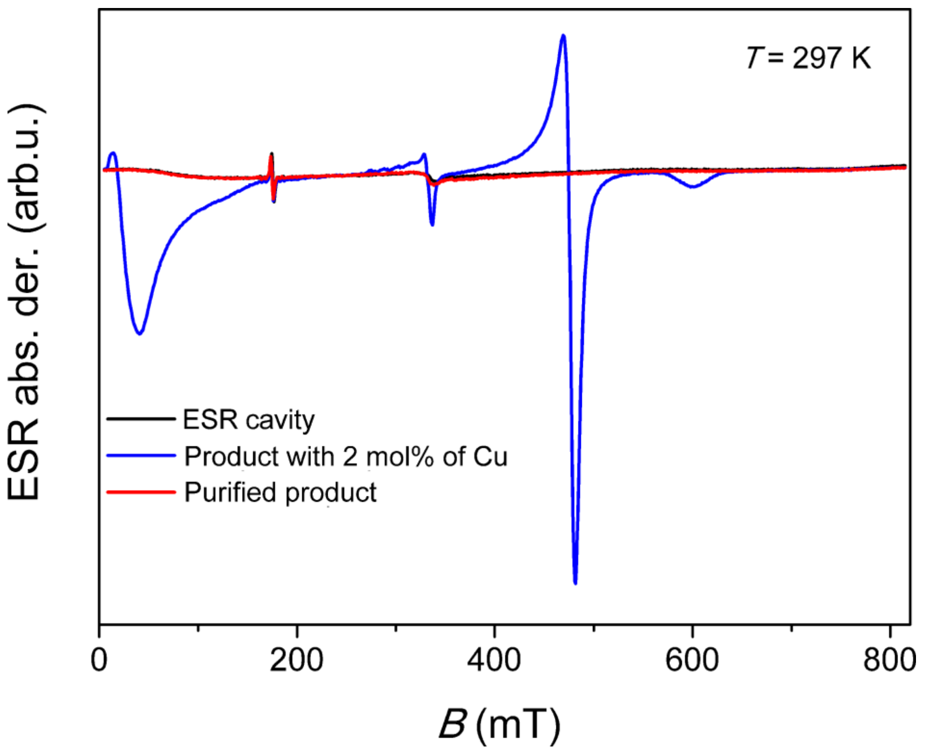

Figure S21. ESR spectra for **5** containing small amounts of copper(II) acetate (blue) and product **5** purified by column chromatography (red).

7. X-ray crystal structure analysis

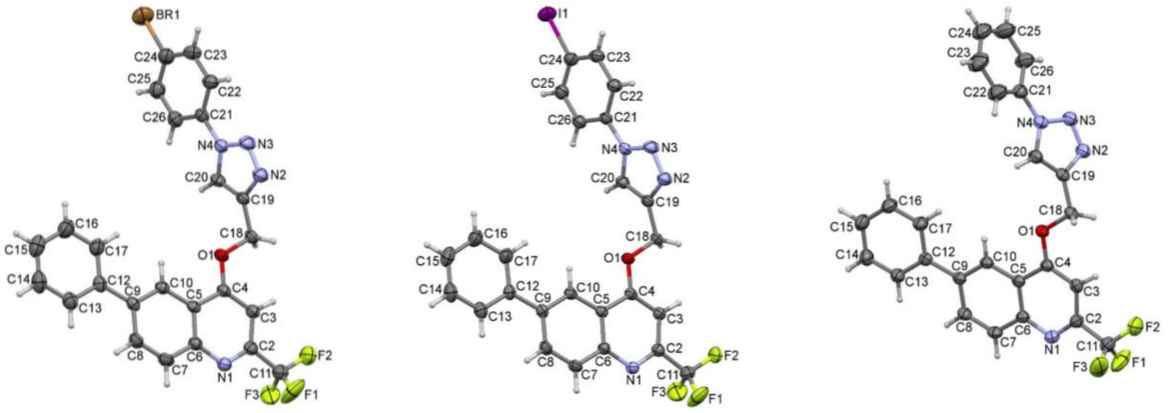

a) b) c)

Figure S22. Molecular structures of **6** (a), **7** (b) and **8** (c), with the atom-numbering schemes. Displacement ellipsoids for non-hydrogen atoms are drawn at the 30% probability level. Only major component of disordered fluorine atoms is presented.

The phenyl ring C12–C17 in **5**–**8** is almost coplanar to the ring to which it is attached; the dihedral angles lie in the range from 1.6 to 2.9° (Table S1). Furthermore, in **5**–**7**, the triazole ring is parallel with the C21–C26 phenyl ring [dihedral angles 2.9 to 5.7°], whereas in **8** it forms an angle of ca. 18°. Finally, the angle between the triazole ring and the N1,C2–C6 ring range from 65.6 to 70.1°, Table S1.

Table S2. Selected dihedral and torsion angles in **5**–**8**

Dihedral / Torsion Angle	**5**	**6**	**7**	**8**
C5–C10 / C12–C17	1.81(10)	2.90(19)	1.58(16)	2.85(9)
N2–N4,C19,C20 / C21–C26	2.89(10)	3.0(2)	5.74(16)	18.39(13)
N2–N4,C19,C20 / N1,C2–C6	68.76(9)	69.71(18)	70.11(14)	65.64(9)
C3–C4–O1–C18	–1.3(2)	–0.8(5)	–1.7(4)	4.2(2)
C4–O1–C18–C19	–173.68(13)	–175.8(3)	–175.4(2)	169.17(14)
O1–C18–C19–N2	110.74(17)	110.9(3)	110.9(3)	–115.76(18)

Table S3. Intermolecular hydrogen-bonding geometries in **5**–**8**

	D–H···A	D–H (Å)	H···A (Å)	D···A (Å)	D–H···A (°)	Symmetry codes
**5**	C20–H20···N1	0.93	2.39	3.275(2)	158	2–x, 1–y, 1–z
	C18–H18A···*Cg*1[a]	0.97	2.76	3.6610(18)	155	2–x, 1–y, 1–z
	C26–H26···*Cg*2[a]	0.93	2.96	3.801(3)	151	x, –1+y, z
**6**	C20–H20···N1	0.93	2.35	3.252(5)	162	1–x, 2–y, 1–z
	C18-H18A···*Cg*1[a]	0.97	2.80	3.686(4)	152	1–x, 2–y, 1–z
**7**	C20–H20···N1	0.93	2.36	3.269(4)	164	1–x, 2–y, 1–z
	C23–H23···F2	0.93	2.55	2.994(5)	110	1–x, 1–y, 1–z
	C18–H18A···*Cg*1[a]	0.97	2.82	3.694(3)	150	1–x, 2–y, 1–z
**8**	C8–H8···N2	0.93	2.74	3.516(2)	142	1–x, –y, 1–z
	C20–H20···N1	0.93	2.64	3.436(2)	144	–x, –y, 1–z
	C15–H15···F1	0.93	2.53	3.262(3)	136	1+x, y, –1+z
	C25–H25···F3	0.93	2.65	3.395(5)	138	1+x, 1+y, –1+z
	C18–H18B···*Cg*1[a]	0.97	2.81	3.7329(18)	160	–x, –y, 1–z
	C26–H26···*Cg*2[a]	0.93	2.89	3.772(3)	159	x, 1+y, z

[a] *Cg*1 and *Cg*2 are centroids of the C5–C10 and C12–C17 rings.

Table S4. π···π interactions geometries

	Ring 1 / Ring 2	Centroids separation (Å)	Interplanar spacing (Å)	Ring inclination (°)	Centroid-centroid offset (Å)	Symmetry codes
**5**	C21–C26 / C21–C26	3.7782(13)	3.5800(9)	0.00(11)	1.208	1–x, –y, 2–z
**6**	C21–C26 / C21–C26	3.832(2)	3.6078(16)	0.00(19)	1.292	1–x, 1–y, –z
**7**	C21–C26 / C21–C26	3.9522(17)	3.6521(13)	0.02(15)	1.511	1–x, 1–y, –z
**8**	C21–C26 / C21–C26	3.9812(18)	3.9670(13)	0.00(15)	0.336	1–x, 1–y, –z

One C−H···π interaction in **5**, C18···*Cg*1, also participates in the dimer formation, while the second one, C26···*Cg*2, forms chains of dimers (Figure S12). The C21–C26 rings of neighbouring molecules are mutually parallel (Table S3) so that one π···π interaction extends chain of dimers into a two-dimensional network (Figure S13).

The main hydrogen-bonded motif in **5**, hydrogen-bonded dimer, was also observed in **6** (Figure S14a). Interestingly, the C26···*Cg*2 interaction present in **5** that links the dimers is missing in this structure. One π····π interaction established between the phenyl rings containing the halogen substituent links the dimers, so that final supramolecular structure of **6** is one-dimensional chain of dimers (Figure S14b and Table S3).

The main hydrogen-bonding motif in **5** and **6** is maintained also in the crystal structure of **7**. Compared to previous two structures one additional supramolecular interaction is present, C–H···F, which forms chain of dimers (Figure S15a).

Besides the C20···N1 hydrogen bond and the C18···*Cg*1 interaction present in **57** and the C26···*Cg*2 interaction present in **5**, structure of **8** contains also one additional C–H···N hydrogen bond and two C–H···F hydrogen bonds (Figure S15b). All these non-covalent interactions form a three-dimensional network (Figure S17). These completely different hydrogen-bonding pattern, and in particular presence of C25···F3 hydrogen bond, could be a reason for a slightly different orientation of the C21−C26 phenyl ring towards the triazole ring in **8** compared to **5**–**7**.

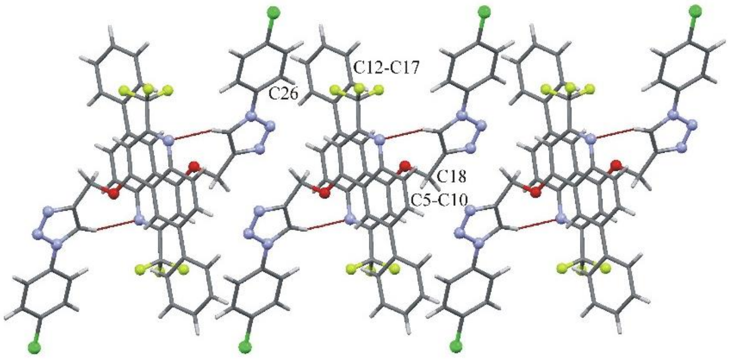

Figure S23. Capped stick representation of **5**, showing chains of dimers formed by one C−H···π interaction. Nitrogen, oxygen and halogen atoms are presented in ball and stick style. Only the major component of disordered fluorine atoms is presented.

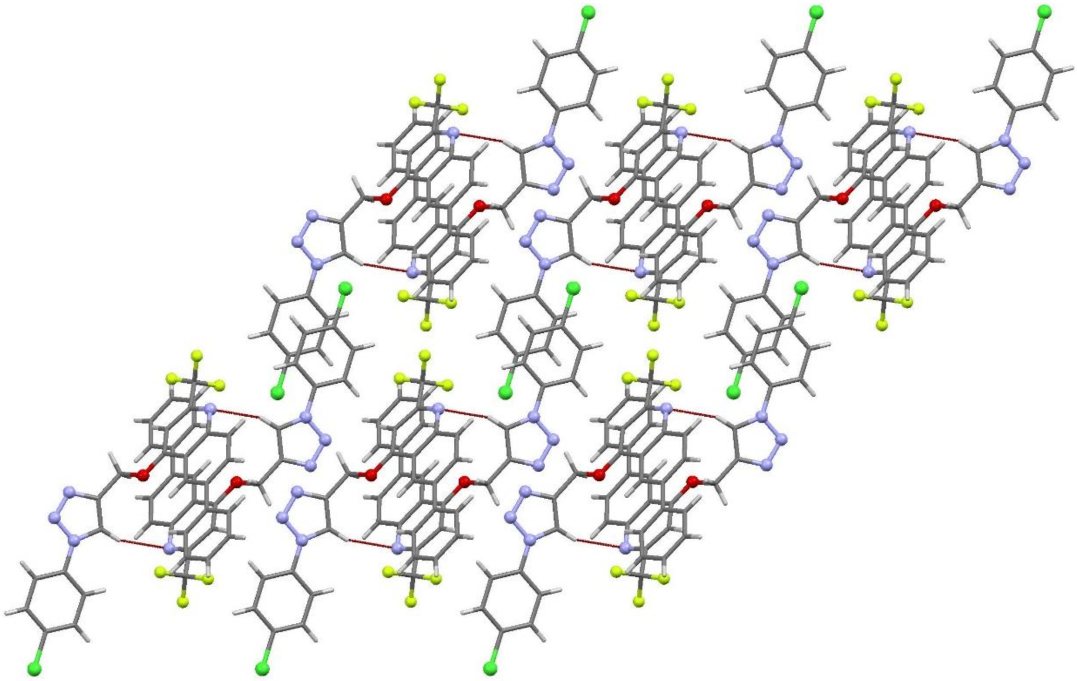

Figure S24. Capped stick representation of **5**, showing one π···π interaction that links chains of dimers into two-dimensional network. Nitrogen, oxygen and halogen atoms are presented in ball and stick style. Only the major component of disordered fluorine atoms is presented.

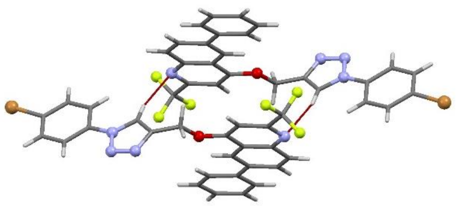

a)

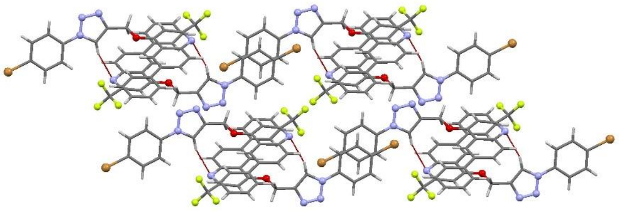

b)

Figure S25. Capped stick representation of **6**, showing the dimer formed by C–H···N hydrogen bond (a) and chains of dimers formed by one π···π interaction (b). Nitrogen, oxygen and halogen atoms are presented in ball and stick style. Only the major component of disordered fluorine atoms is presented.

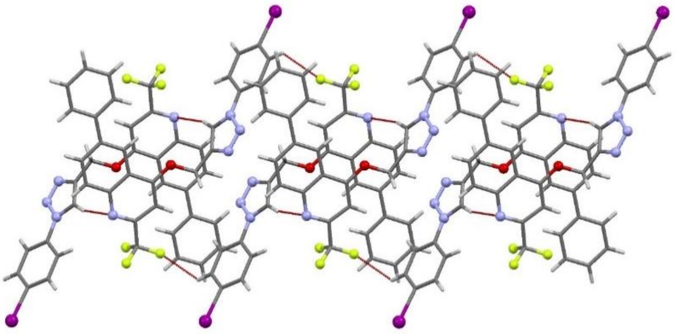

a)

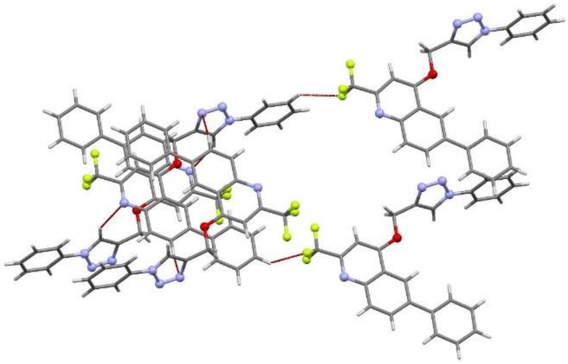

b)

Figure S26. (a) Capped stick representation of **7**, showing chains of dimers formed by C−H···F hydrogen bonds. (b) Capped stick representation of **8**, showing C–H···N and C–H···F hydrogen bonds. Nitrogen, oxygen and halogen atoms are presented in ball and stick style. Only the major component of disordered fluorine atoms is presented.

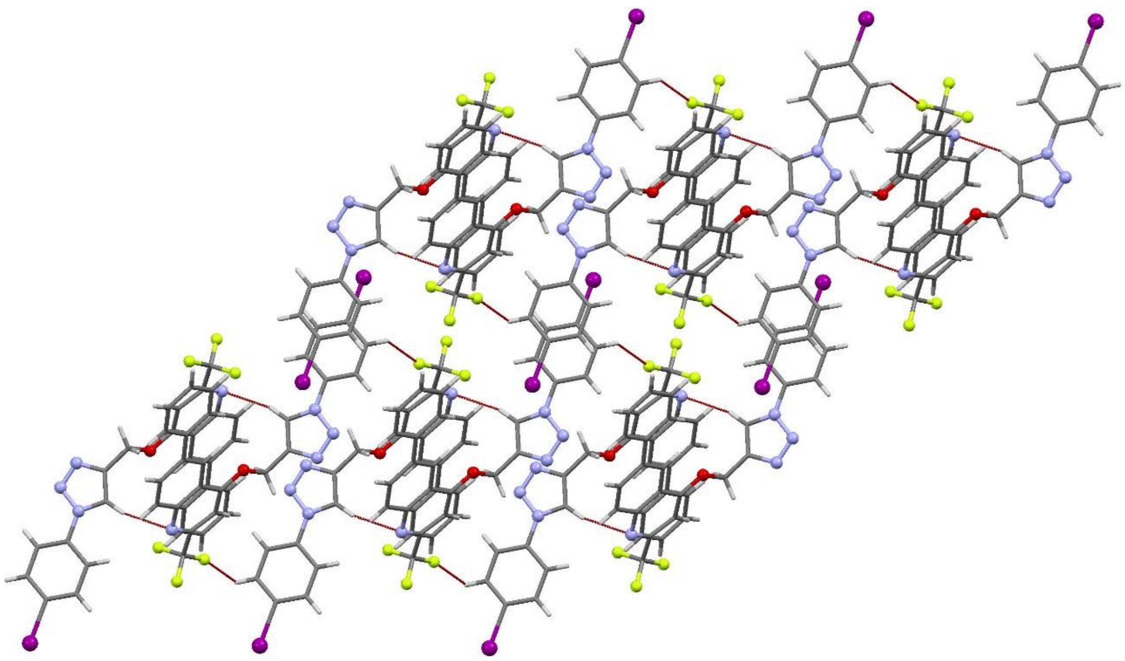

Figure S27. Capped stick representation of **7**, showing one π···π interaction that links chains of dimers into two-dimensional network. Nitrogen, oxygen and halogen atoms are presented in ball and stick style. Only the major component of disordered fluorine atoms is presented.

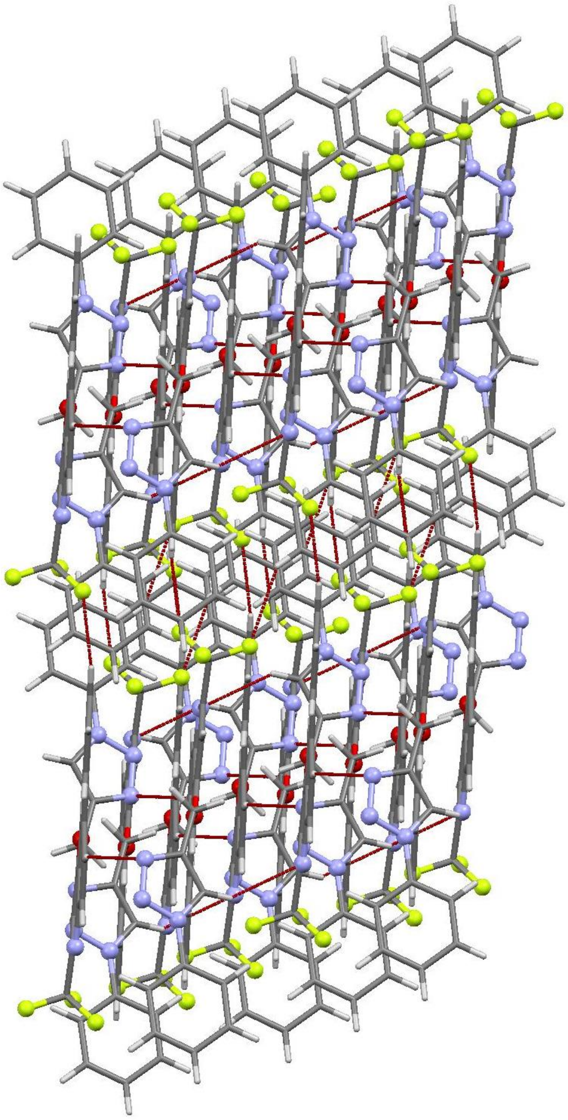

Figure S28. Capped stick representation of **8**, showing three-dimensional network formed by C–H···N and C–H···F hydrogen bonds, one C−H···π interaction and one π···π interaction. Nitrogen, oxygen and halogen atoms are presented in ball and stick style. Only the major component of disordered fluorine atoms is presented.
